# To what extent do nurses use research in clinical practice? A systematic review

**DOI:** 10.1186/1748-5908-6-21

**Published:** 2011-03-17

**Authors:** Janet E Squires, Alison M Hutchinson, Anne-Marie Boström, Hannah M O'Rourke, Sandra J Cobban, Carole A Estabrooks

**Affiliations:** 1 Clinical Epidemiology Program, Ottawa Hospital Research Institute, Ottawa, Canada; 2 Faculty of Nursing, Deakin University, and Cabrini-Deakin Centre for Nursing Research, Cabrini Institute, Cabrini Health, Melbourne, Australia; 3 Department of Neurobiology, Care Sciences and Science, Division of Nursing, Karolinska Institutet, Stockholm, Sweden; 4 Faculty of Nursing, University of Alberta, Edmonton, Alberta, Canada; 5 Faculty of Medicine and Dentistry, University of Alberta, Edmonton, Alberta, Canada

## Abstract

**Background:**

In the past forty years, many gains have been made in our understanding of the concept of research utilization. While numerous studies exist on professional nurses' use of research in practice, no attempt has been made to systematically evaluate and synthesize this body of literature with respect to the extent to which nurses use research in their clinical practice. The objective of this study was to systematically identify and analyze the available evidence related to the extent to which nurses use research findings in practice.

**Methods:**

This study was a systematic review of published and grey literature. The search strategy included 13 online bibliographic databases: Cochrane Database of Systematic Reviews, Cochrane Central Register of Controlled Trials, MEDLINE, CINAHL, EMBASE, HAPI, Web of Science, SCOPUS, OCLC Papers First, OCLC WorldCat, ABI Inform, Sociological Abstracts, and Dissertation Abstracts. The inclusion criteria consisted of primary research reports that assess professional nurses' use of research in practice, written in the English or Scandinavian languages. Extent of research use was determined by assigning research use scores reported in each article to one of four quartiles: low, moderate-low, moderate-high, or high.

**Results:**

Following removal of duplicate citations, a total of 12,418 titles were identified through database searches, of which 133 articles were retrieved. Of the articles retrieved, 55 satisfied the inclusion criteria. The 55 final reports included cross-sectional/survey (n = 51) and quasi-experimental (n = 4) designs. A sensitivity analysis, comparing findings from all reports with those rated moderate (moderate-weak and moderate-strong) and strong quality, did not show significant differences. In a majority of the articles identified (n = 38, 69%), nurses reported moderate-high research use.

**Conclusions:**

According to this review, nurses' reported use of research is moderate-high and has remained relatively consistent over time until the early 2000's. This finding, however, may paint an overly optimistic picture of the extent to which nurses use research in their practice given the methodological problems inherent in the majority of studies. There is a clear need for the development of standard measures of research use and robust well-designed studies examining nurses' use of research and its impact on patient outcomes. The relatively unchanged self-reports of moderate-high research use by nurses is troubling given that over 40 years have elapsed since the first studies in this review were conducted and the increasing emphasis in the past 15 years on evidence-based practice. More troubling is the absence of studies in which attempts are made to assess the effects of varying levels of research use on patient outcomes.

## Background

Scholars have expressed long-held concerns about whether nurses' practice is in accordance with the best available scientific evidence [1-9]. The disparity between the findings of research evidence and actual practice is frequently referred to as the research-practice gap [6,10-12]. Despite increasing quantities of, and more convenient access to, clinically relevant research, the slow and haphazard uptake or failure to adopt such evidence persists. Many examples of the research-practice gap have been highlighted in the nursing literature over the past thirty years [13-15]. However, most of the evidence is anecdotal, highlighting the difficulties surrounding attempts to measure whether or not a practice is based on research [16]. Bostrom and Wise [17] suggested that the research-practice gap is in the order of ten to fifteen years, while Landrum [18] proposed that an eight to thirty year time lag exists between publication and adoption of research findings.

Concerns about this gap are related to widely held assumptions that patients who receive evidence-based care achieve better outcomes. There is some evidence in support of this assumption [19-22]. However, because such evidence has often resulted from studies conducted under research conditions, Estabrooks [6] recommends caution when drawing this conclusion. Estabrooks argued that we do not know whether and to what extent nurses adapt research findings according to the circumstances and context in which they practice, and consequently, the effectiveness of nursing interventions under such conditions is largely unknown. The gold standard of evidence in healthcare intervention (including interventions to promote research utilization) research is commonly held to be the prospective randomized controlled trial (RCT). RCTs can be either explanatory or pragmatic in nature. Explanatory trials test whether an intervention is efficacious (*i.e.*, whether it is beneficial in an 'ideal' situation), while pragmatic trials measure effectiveness (the degree of beneficial effect in 'real' practice). Hence, the pragmatic trial is more often a reflection of the 'real world' and therefore, if used, would address the concerns raised by Estabrooks [6] about the extent to which nurses adapt research findings according to the circumstances and context in which they practice.

Research utilization is defined by Estabrooks and colleagues [23] as 'that process by which specific research-based knowledge (science) is implemented in practice.' It is a complex and multi-facetted construct, as evidenced by the multiple and diverse conceptualizations that abound the literature. For instance, while some scholars define research utilization as a general or omnibus construct [24,25], others describe it as the use of specific research-based findings or practices [26,27]. Within these views, research utilization is often conceptualized further as a variable (or discrete event) [24,28,29] or a process (as consisting of a number of consecutive steps or stages) [26,30]. Some scholars, in addition to using a variable conceptualization, also propose several different kinds of research utilization exist; instrumental, conceptual, and persuasive uses of research have been described [31-33]. Instrumental utilization refers to the concrete application of specific knowledge to practice; conceptual utilization refers to a change in thinking, but not necessarily behavior, in response to research findings; and persuasive utilization refers to the use of certain knowledge to persuade others regarding a predetermined position [23,34]. Estabrooks [29] embarked on a study to explore and provide some empirical support for a conceptual structure of research utilization and concluded that 'instrumental, conceptual, and persuasive research utilization exist and that a global measure of research utilization may be defensible.' She argued, however, that we have little understanding of the correct measures of research utilization, and that the most common methods employed measure only instrumental research use [35]. She further contended that in failing to measure conceptual and persuasive research utilization, the findings of such studies underestimate nurses' overall research utilization (*i.e.*, the use of any kind of research in any way in clinical practice) [29].

Several published reviews have identified the complexity of, and challenges associated with, integrating research evidence into practice [36,37]. A number of factors related to characteristics of the evidence, the individual practitioner, and the context in which care is delivered have been identified as being influential in the translation of research to practice. A 2003 review [38] reported six categories of potential individual determinants of research utilization: beliefs and attitudes, involvement in research activities, information seeking, professional characteristics, education, and other socio-economic factors. More recently, Meijers *et al. *[39] examined the relationships between characteristics of organizational context and research use. They reported statistically significant relationships between research use and six contextual factors: the role of the nurse, multi-faceted access to resources, organizational climate, multifaceted support, time for research activities, and provision of education. Despite these findings, attempts to measure the extent of research use have not captured the complexity of the phenomenon.

In addition to the reviews identified above, reviews exist on studies measuring research use by nurses [23], interventions designed to increase nurses' use of research [40], and instruments used to measure nurses' attitudes towards research use [41]. However, we could locate no reviews on the question of whether, and to what extent, nurses use research. The purpose of the systematic review reported in this paper was therefore to examine existing evidence on the extent to which nurses use research in clinical practice and by so doing, contribute to the body of work assessing the 'state of the science' in this field.

## Methods

### Inclusion criteria

#### Types of studies

Experimental (intervention) and non-experimental designs that examined the use of research by nurses in clinical practice were eligible for inclusion. An experiment was defined as 'a study in which an intervention is deliberately introduced to observed its effects' [42]. Experimental designs include RCTs, clinical trials, and quasi-experimental (*e.g.*, pre/post test designs) studies. Non-experimental designs refer to observational studies (*e.g.*, cohort, case-control, cross-sectional) [42]. Case reports and non-systematic/narrative literature reviews were excluded. Studies were limited to those published in English and Scandinavian languages -- the languages represented on the research team. There were no restrictions on the basis of country of origin or publication date.

#### Types of participants and outcomes

Studies that examined nurses' use of research in clinical practice were considered for inclusion. A nurse was defined as a professional who provides patient care in a clinical setting -- *e.g.*, registered nurse (RN), registered psychiatric nurse (RPN), licensed practical nurse (LPN). The outcome of interest was use of research findings. Research, in this study, was defined as information that is empirically and systematically derived. The research findings could be reported in a primary research article, review/synthesis report, or protocol. Measures of research use needed to be expressed quantitatively.

We excluded articles that reported on nurses' adherence to clinical practice guidelines, the rationale being that clinical practice guidelines can be based on non-research evidence (*e.g.*, expert opinion). We did not have the capacity to review the evidence base for recommendations provided in each guideline reported in the literature. We did however include nurses' use of protocols where the research-base of the protocol was made explicit in the research report. It is also possible that studies assessing research utilization using omnibus (general) questions may include nurses' use of guidelines if the nurse answered with a specific guideline in mind. We also excluded articles that reported on: predictors or barriers to research utilization if they did not also report on nurses' use of research in their practice; nurses' use of one specific research-based practice if the purpose of the study was not to examine nurses' use of research in practice; and studies in which research use of healthcare professionals other than nurses were included if a separate analysis of nurses' use of research was not provided or could not be extracted. We also excluded articles where a quantitative measure of the extent of research use was not provided or could not be derived from the data reported.

### Search strategy for identification of studies

The search strategy for this review was developed in consultation with a health sciences librarian. We searched the following bibliographic databases: Cochrane Database of Systematic Reviews, Cochrane Central Register of Controlled Trials, CINAHL, MEDLINE, EMBASE, HAPI, Web of Science, SCOPUS, OCLC PapersFirst, OCLC WorldCat, ABI Inform, Sociological Abstracts, and Dissertation Abstracts. Key words and subject headings related to research use and known instruments to assess research use in nurses were identified prior to initiating the search. The derivatives of the search terms were captured with the use of truncation symbols appropriate to the respective databases searched. Due to the differences in meaning between the terms 'research utilization' and 'evidence-based practice' (*i.e.*, evidence-based practice is sometimes used as a broader concept which can incorporate forms of knowledge other than research) we decided, in consultation with a health sciences librarian, to exclude the term 'evidence-based practice' from the search strategy. See Table 1 for a summary of the search strategy.

**Table 1:** Search Strategy

Database	Edition	No. Articles
Cochrane database of Systematic Reviews	Through to 1^st ^quarter 2008	0

Cochrane Central Register of Controlled Trials	Through to 1^st ^quarter 2008	0

CINAHL	Through to May 7, 2007	3,130

MEDLINE	Through to May 7, 2007	3,842

EMBASE	Through to May 7, 2007	2,881

HAPI	Through to May 7, 2007	7,212

Web of Science	Through to May 7, 2007	2,616

SCOPUS	Through to May 7, 2007	2,080

OCLC PapersFirst	Through to May 9, 2007	62

OCLC WorldCat	Through to May 9, 2007	269

ABI Inform	Through to May, 2007	641

Sociological Abstracts	Through to May 9, 2007	332

Dissertation Abstracts	Through to February 9, 2008	1,300

Subtotal		24,365

Duplicates removed		- 11,947

**Total**		**12,418**

**Search terms**: (1) Research uptake, (2) research use, (3) uptake of research, (4) innovation diffusion, (5) diffusion of innovation(s), (6) dissemination of innovation(s), (7) research utili$ation, (8)research dissemination, (9) dissemination of research, (10) diffusion of research, (11) research transfer, (12) research implementation, (13) knowledge transfer, (14) knowledge uptake **OR **(15) nursing practice questionnaire, (16) research utilization survey, (17) Edmonton research orientation **AND **(18) survey, (19) survey*, (20) questionnaire*, (21) instrument*, (22) scale*, (23) reliability, (24) validity, (25) validation, (26) reproducib*, (27) benchmark*, (28) measur*, (29) evaluat*, (30) assess*

*Map subheadings and specific words truncations were used per each database

### Study identification

Two team members (JES and HMO) independently screened the titles and abstracts of the 12,418 citations identified by the search strategy noted in Table 1 to identify potentially relevant studies. Full text copies were retrieved of all citations identified as: having potential relevance to the objective of the review and where there was insufficient information to make a decision as to relevance (n = 133). Two reviewers (JES and HMO) then independently assessed all retrieved articles for relevance. A total of 55 articles were retained. All discrepancies with respect to relevance were resolved through consensus.

### Quality assessment

All included articles (n = 55) were independently assessed for methodological quality by two reviewers (two of JES, AMH, AMB, HMO). To assess methodological quality we adapted two previously used tools. Disagreements in quality assessment were resolved through consensus.

The first tool, the Estabrooks' Quality Assessment and Validity Tool for Cross-Sectional Studies, originally developed based on Cochrane guidelines (in existence in 2001) and the medical literature [43,44], has been used in other systematic reviews by our group [38,45]. The tool contains a maximum of 16 points and assesses studies in three core areas: sampling, measurement, and statistical analysis. To derive a final quality score for each article, we divided the total points scored by the total points possible (16 -- the number of points not applicable for the article). Each study was then classified as weak (≤0.50), moderate-weak (0.51 to 0.65), moderate-strong (0.66 to 0.79), or strong (≥0.80). This rating system was used in a recent review [45] and is based on a scoring system developed by de Vet *et al. *[46]. This tool was used to assess the methodological quality of all cross-sectional studies included in the review (n = 51). That is, all studies providing a descriptive snapshot at one point in time of the extent to which nurses use research in practice.

The second quality assessment tool used in this review was the Quality Assessment Tool for Quantitative Studies (http://www.city.hamilton.on.ca/phcs/EPHPP/), developed by the Effective Public Health Practice Project, Canada. This tool has been judged suitable to be used in systematic reviews of effectiveness (measuring interventions) [47], and been shown to have content and construct validity [48]. The tool assesses studies on the basis of six areas; the six areas are selection bias, study design, confounders, blinding, data collection methods, and withdrawals/drop-outs. Each article is scored as weak, moderate, or strong in each of these six areas according to preset criteria within the tool. The tool developers do not provide a means for calculating an overall quality score. However, in order to compare the quality scores for each included article that used an intervention design (assessed with this tool) to the included articles that used cross-sectional designs (assessed with Estabrooks' Quality Assessment and Validity Tool described above), we derived an overall quality score for each article. To derive this score, we assigned values of 1, 2, and 3 to the categorizations of weak, moderate, and strong respectively. A final quality score for each article was then obtained by dividing the summative score obtained by the total amount of points possible. Each study was classified as weak (1 to 1.5), moderate-weak (1.6 to 2.0), moderate-strong (2.1 to 2.5), or strong (>2.5) by applying the same categorization system used (and published) in the cross-sectional tool. The Quality Assessment Tool for Quantitative Studies tool was used to assess all intervention studies included in the review (n = 4). That is, all studies testing an intervention to improve nurses' use of research.

### Data extraction

Two reviewers performed data extraction on all included articles; one reviewer extracted data, which was then checked for accuracy by a second reviewer. Data were extracted on: study design, country, sample and subject characteristics, setting, measure of research use, reliability and validity, main finings with respect to use of research, and the intervention (where applicable). For the four intervention studies, data were extracted on both pre- and post-research use scores. All disagreements in data extraction were resolved through consensus.

### Data analysis

The use of many different measures of research use across different healthcare contexts prevented us from performing a meta-analysis. Therefore, the findings from the review are presented in narrative form. That is, we synthesized the extracted data descriptively, according to the type of measure used to assess nurses' use of research as follows: Nurses Practice Questionnaire (NPQ), Research Utilization Questionnaire (RUQ), other multi-item measures, and single-item measures. A sensitivity analysis, comparing findings from all reports with those rated moderate (moderate-weak and moderate-strong) and strong quality, was performed to assess the impact of methodological quality on the review findings; no significant differences were noted. Therefore, we have elected to report findings from all 55 included reports.

To determine the extent to which nurses use research in their practice across all included reports, we categorized the findings from each study onto a common metric: low research use, moderate-low research use, moderate-high research use, and high research use (these findings are summarized in Table 2). We did this by creating equal quartiles for each of the different scoring systems used in the selected articles. For example, 12 articles [5,26,27,33,49-56] used the NPQ to measure nurses' use of research. The NPQ provides a total innovation adoption behavior (TIAB) score for each nurse (which represents their overall research use) based on responses to a series of questions about specific research-based practices. The TIAB score can range from 0 to 4, with 4 indicating maximum research use; by equally dividing the possible range of scores into quartiles we were able to categorize the extent to which nurses use research in their clinical practice as follows: low (0 to 0.99), moderate-low (1.00 to 1.99), moderate-high (2.00 to 2.99), and high (3.0 to 4.00). We used similar processes to create quartiles for the research use scores provided in all 55 included articles. For the four intervention studies, we used the pretest research utilization scores to calculate quartiles. The pretest scores were used for two reasons: posttest scores could be lower or higher due to chance, and posttest scores may not be sustained overtime. Therefore, we hypothesized the pretest scores would be a more accurate reflection of the extent to which nurses use research in their clinical practice. A description of the processes used to calculate extent of research use in each of the included articles can be found in Additional Files 1, 2, 3 and 4. We elected to use this (quartile) method to synthesize the findings because it allowed us to compare all 55 included reports to provide an overall conclusion on the extent to which nurses use research use in practice. While a few instruments (*e.g.*, NPQ, RUQ) were used in multiple studies, we elected not to calculate additional summary statistics for these instruments because: it would not progress our overall aim of determining the extent to which nurses use research overall, several of the multi-use instruments were modified significantly, limiting ability to combine scores, and there was little variability in scores between studies using the same instrument, and therefore, it would not provide added value.

**Table 2:** Summary of Findings

Instrument	First Author, Year	Quartile 1	Quartile 2	Quartile 3	Quartile 4
		*Low Use*	*Moderate-Low Use*	*Moderate-High Use*	*High Use*
**NPQ **(n = 12 articles)	Brett, 1987 [26]			X	
	
	Brett, 1989 [50]			X	
	
	Coyle, 1990 [51]		X		
	
	Barta, 1995 [52]			X	
	
	Michel, 1995 [5]			X	
	
	Berggren, 1996 [33]			X	
	
	Rutledge, 1996 [53]				X
	
	Thompson, 1997 [54]			X	
	
	Rodgers, 2000 [27]			X	
	
	Rodgers, 2000 [55]			X	
	
	Carlson, 2006 [62]			X	
	
	Squires, 2007 [56]			X	

**RUQ **(n = 10 articles)	Champion, 1989 [63]			X	
	
	Lacey, 1994 [71]			X	
	
	Prin, 1997 [83]			X	
	
	Hatcher, 1997 [68]			X	
	
	Hansen, 1999 [67]			X	
	
	Humphris, 1999 [69]			X	
	
	Tranmer, 2002 [91]			X	
	
	Wallin, 2003 [95]			X	
	
	McCloskey, 2005 [75]		X		
	
	Nash, 2005 [57]		X		

**Other Multi-Item Measures **(n = 5 articles)	Pelz, 1981 [90]		X		
	
	Varcoe, 1995 [87]		X (general use)	X (specific research findings)	
	
	Stiefel, 1996 [85]			X	
	
	McCleary, 2002 [73]			X	
	
	McCleary, 2003 [74]			X	

**Single items**	Bostrom, 1993 [60]	X			
*Past, Present, Future Use *(n = 4 articles)					
	
	Rizzuto, 1994 [4]	X			
	
	Butler, 1995 [59]		X (staff nurses)	X (leadership nurses)	
	
	Brown, 1997 [61]		X		

**Single items**	Parahoo, 1998 [24]		X (specific research findings)	X (general use)	
*Parahoo Measure *(n = 7 articles)					
	
	Parahoo, 1999 [78]		X		
	
	Parahoo, 1999b [79]			X	
	
	Parahoo, 2000 [80]		X		
	
	Parahoo, 2001 [81]			X	
	
	Valizadeh, 2003 [86]		X		
	
	Veeramah, 2004 [88]			X	

**Single items**	Estabrooks, 1999 [29]		PRU	IRU, CRU, ORU	
*Estabrooks Measure *(n = 6 articles)					
	
	Profetto-McGrath, 2003 [84]			PRU, ORU	CRU
	
	Milner, 2005 [76]		PRU (staff)	IRU (staff, managers) CRU (staff, managers) ORU (staff, managers) PRU (educators, managers)	IRU (educators) CRU (educators) ORU (educators)
	
	Kenny, 2005 [70]		PRU	IRU, CRU, ORU	
	
	Estabrooks 2007 [66]			IRU, ORU	
	
	Connor, 2007 [64]		CRU	IRU, ORU	PRU

**Single items**	Linde, 1989 [89]	X^1^	X^2^		
*Other *(n = 11 articles)					
	
	Walczak, 1994 [96]		X		
	
	Pettengill, 1994 [82]		X^3^	X^4^	
	
	Veeramah, 1995 [88]			X	
	
	Youngstrom, 1996 [98]			X	
	
	Wright, 1996 [97]			X	
	
	Logsdon, 1998 [72]		X		
	
	Davies, 1999 [99]		X^5^	X^5^	X^5^
	
	Tsai, 2000 [58]			X	
	
	Tsai, 2003 [92]		X		
	
	Niederhauser, 2005 [77]				X

IRU = instrumental research use; CRU-conceptual research use; PRU = persuasive research use; ORU = overall research use

^1 ^= Item: Discontinue practice based on research

^2 ^= Items: (1) transfer knowledge from research; (2) use new research-based activity

^3 ^= Use of nursing research

^4 ^= Use of non-nursing research

^5 ^= 23 practices assessed, extent of research use depends on the practice assessed

## Results

### Description of studies

**Figure 1:**
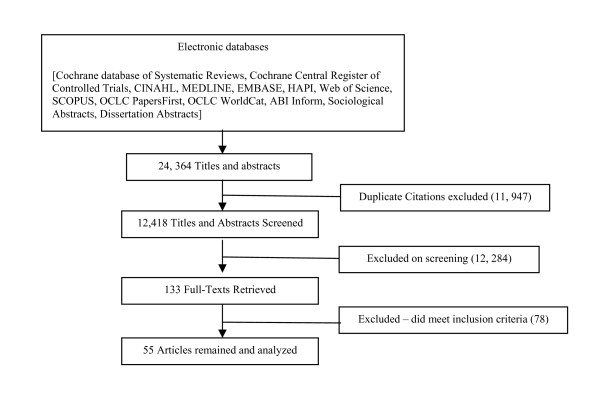
**Search and Retrieval Process**.

The database search yielded 12,418 unique (after removal of duplicate articles) titles and/or abstracts. Of these, 133 were identified as potentially relevant and were retrieved in full text. From the 133 retrieved articles, 78 did not meet our inclusion criteria (see Additional File 5). This resulted in a final sample of 55 articles (see Figure 1) representing 47 individual studies. Of the final 55 articles, the majority (n = 51) reported a cross-sectional survey design [4,5,9,24,26,27,29,33,50-88], while the remainder (n = 4) used a quasi-experimental design [89-92].

**Figure 2:**
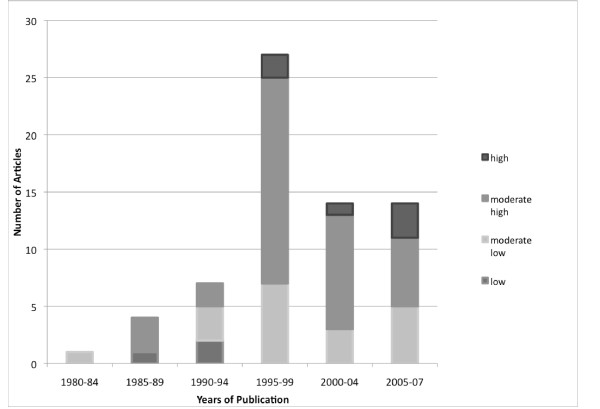
**Extent of research use by year of publication (n = 55 articles)**.

The majority of articles examine research use by nurses in North America (n = 39, 71%) followed by Europe (n = 12, 22%), Asia (n = 3, 5%) and Oceania (n = 1, 2%). Most studies were conducted in acute care (hospital) settings (n = 44, 80%) followed by multiple settings (*e.g.*, sampling through a register) (n = 9, 16%), educational programs (n = 1, 2%) and nursing homes (n = 1, 2%). With respect to year of publication, the first report included in this review was published in 1981 [90]. Earlier reports on research use in nurses dating back to the 1970's [93] were excluded from this review because their purpose was to measure the use of a practice and not the concept of research use *per se*. There has been a trend of increased published reports on nurses' use of research in the past decade, with 38 (69%) of the 55 reports in this review being published between 1996 and 2007. The number of published articles peaked in 1995 to 1999, followed by a gradual fall in the early 2000's (Figure 2); this may be due, in part, to a shift in focus by some researchers away from research utilization and towards evidence-based practice. Examination of the articles in chronological order reveals that early studies found low, moderate-low, or moderate-high research use only (Figure 2). The first study in which high research use was found was published in 1996 [53] and since then moderate-low, moderate-high, and high reports of research use have been published, with no studies falling into the low research use category. Characteristics of the 55 included articles can be found in Additional Files 1, 2, 3, and 4.

### Methodological quality of studies

Methodological quality of the 55 included articles is presented in Additional File 6. Of the included reports, 3 (5%) were rated as strong [27,76,86], 12 (22%) as moderate-strong [5,26,29,50-52,54-56,66,91,94], 23 (42%) as moderate-weak [9,24,33,49,53,61,68-70,72,73,77,78,80,81,83,84,87-89,92,95,96], and 17 (31%) as weak [4,57,59,60,63-65,67,71,74,75,79,82,85,90,97,98]. Discrepancies in quality assessment between reviewers related primarily to representativeness of the sample, treatment of missing data, and appropriateness of statistical tests, and were settled through consensus. In the majority of articles, the research use measures employed were reported to be reliable and valid. However, stringent assessments were not performed. Reliability (of the multi-item measures) was limited to tests of internal consistency (Cronbach's alpha) while assessment of validity (of both multi and single items) was predominantly limited to content validity and in many cases was 'assumed' based on a review of the literature and/or a statement from the index (first) study of the measure stating it was a valid instrument. Other common methodological weaknesses included: low response rates in cross-sectional studies (acceptable response rates (>50%) were only achieved by 42% (n = 21) of the cross sectional studies), and minimal use of control for confounding factors in the study design and/or analysis.

### Study outcome: extent of research utilization

Because nurses' use of research was assessed with several different measures of research use, we have grouped findings on the extent of research use according to the class of instrument used in its assessment. A summary of these findings is presented in Table 2. Within each instrument group we also emphasize the extent of research use according to whether it was measured as research use in general, the use of specific research findings, or according to a kind of research use -- *i.e.*, instrumental, conceptual, persuasive, or overall research use.

### Nurses practice questionnaire

Twelve articles (ten studies) [5,26,27,33,49-56] assessed nurses' use of research using the NPQ. The NPQ consists of brief descriptions of a set of specific nursing practice innovations (research-based practices). Six questions, which measure the nurse's stage of innovation adoption, are posed for each of the nursing practice innovations. Nurses are then classified as being unaware of, aware of, persuaded of, use sometimes, or use always for each of the practices and for all practices overall. While the adoption scores varied slightly by the specific practices assessed in the included studies, overall adoption scores were similar across studies. Nurses in nine [5,26,27,33,50,51,54-56] of the twelve NPQ articles, on average, reported some use of the practices and were classified as being in the 'persuasion' stage of adoption overall, according to the TIAB classification system developed by Brett [26]. With respect to our extent of research use classification, most NPQ articles (10 of 12) [5,26,27,33,49,50,52,54-56] fell in the moderate-high research use category; one study [51] fell in the moderate-low research use category, while another article [53] fell in the high research use category. Characteristics of the twelve articles using the NPQ to assess nurses' research use can be found in Additional File 1.

### Research utilization questionnaire

Ten articles (ten studies) [57,63,67-69,71,75,83,91,95] assessed nurses' use of research using the RUQ. The RUQ, developed by Champion and Leach [63], is a general measure of research use consisting of 42 self-descriptive statements comprising four subscales of which research utilization is one subscale. The research utilization subscale contains ten items, each scored on a 5-point Likert scale, assessing the degree to which nurses' perceive they incorporate research findings into their daily practice. The ten items are then summed and a mean is taken to obtain an overall research use score, with higher values indicating higher levels of research use. Eight [63,67-69,71,83,91,95] of the ten articles reported an overall score indicative of moderate-high research use. The remaining two articles [57,75] reported moderate-low research use scores. Characteristics of the ten articles using the RUQ to assess nurses' research use can be found in Additional File 2.

### Other multi-item measures

We located an additional five articles (four studies) [73,74,85,87,90] using different multi-item measures to assess nurses' use of research. All articles reported moderate research use. One article [90] reported moderate-low research use in general, while three articles [73,74,85] reported moderate-high research use in general. The remaining article [87] in this category assessed both nurses' use of specific research-based practices (with moderate-high research use scores) and their use of research in general (with moderate-low research use scores). Characteristics of the five articles using individual multi-item measures to assess nurses' research use are found in Additional File 3.

### Single-item measures

In 28 published papers (23 studies), investigators used single-item questions to assess nurses' research use. A combination of specific practices and general research use were targeted with the single-item questions. We further categorized the single-item questions as follows: past, present, and future use; Parahoo's measure; Estabrooks' kinds of research use; and other single items. Characteristics of the 28 articles using single-item measures are found in Additional File 4.

### Past, present, and future research use

Four articles (four studies) [4,59-61] assessed the proportion of nurses reporting past use of research in general (more than six months ago), present use of research in general (most recent six months), and/or future (within the next year) intention to use research in general. While intention to use research in the future was reported as moderate-low [4] to high [59], current use was reported as low at 15.9% [4,60] or moderate-low at 30.3% [59]. In all studies, past use was reported slightly higher at 23.4% [60], 24.6% [4], and 52.6% [59], compared to present use. One article, Brown [61], assessed past use without assessing present use and found that the extent of reported use was moderate-high but varied by type of use: past use of research to change practice (66%), and past use for patient care (71%). Similar to previous studies, Brown [61] also found future intention to use research to be very high (86%) (See Additional File 4).

### Parahoo's measure

Seven articles (three studies) [9,24,78-81,86] assessed nurses' use of research in general using a single-item question developed by Parahoo [24]. This question asked nurses to indicate the frequency with which they used research in clinical practice. The majority of nurses (50.0% to 54.7%), regardless of context (setting) or role, reported moderate research use, with three articles [78,80,86] reporting scores indicative of moderate-low research use and four articles [9,24,79,81] reporting scores indicative of moderate-high research use (See Additional File 4).

### Estabrooks' kinds of research use

Six articles (five studies) [25,64,66,70,76,84] assessed nurses' use of four kinds of research use: instrumental, conceptual, persuasive, and overall. Five [25,64,66,70,84] of the six articles used a 7-point frequency scale; mean scores showed that nurses, on average, reported using research on half of their shifts (score of 5). Nurses also commonly reported using research conceptually and overall more frequently than instrumentally and persuasively. For instance, Estabrooks [29] reported mean scores of 5.20, 4.71, 4.36, and 3.60 (on a 7-point scale) for conceptual, overall, instrumental, and persuasive research use, respectively. The remaining study [76] used a 5-point frequency scale and found a similar pattern for staff nurses' use of research. The extent of research use for nurses reporting kinds of research use ranged from moderate-low to high depending on the kind of research use (with conceptual use generally scoring higher) and nurse group (educator groups generally scored higher compared to staff nurses) (See Additional File 4 and Table 2).

### Other single items

Eleven additional articles (eleven studies) [65,72,77,82,88,89,92,94,96-98] assessed nurses' use of research using their own single-item question(s), which have not been used in subsequent studies. Of these studies, one measured use of specific practices [99], while the remainder measured nurses' use of research in general. Findings varied widely from a low extent of research use [89] to a high extent of research use [65,77]. However, most studies reported moderate-low or moderate-high research use, with some studies reporting both levels of moderate use, depending on the item used to assess research use (See Additional File 4).

### Extent of research use in general, use of specific research findings and research use according to kinds

**Figure 3:**
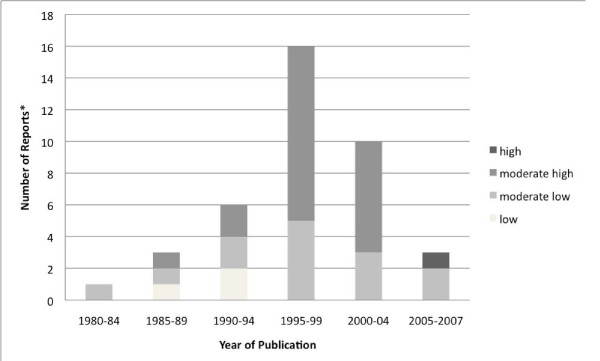
**Extent of research use in general by year of publication (N = 36 articles)**. Three studies (Butler, 1995; Linde, 1989; and Pettingill, 1994) report two levels of research use, either based on responses to separate survey questions or for categories of nurses. Thus, 36 articles account for 39 measures reported in this figure.

The use of research in general was measured in 36 studies, including those in which the RUQ was used [57,63,67-69,71,75,83,91,95], studies that used other multi-item measures [73,74,85,87,90], single-item (past present and future) measures [4,59-61], the single-item Parahoo measure [9,24,78-81,86], and other single-item measures [58,72,77,82,88,89,92,96-98] (Table 2). Research use in general was found to range from low through to high use. Specifically, two studies reported low use [4,60], one study [89] reported low and moderate-low use on the basis of responses to two separate survey items, one study [59] reported moderate-low and moderate-high use dependent on the role of nurses, and one study [77] reported high use. The remainder reported moderate-low (n = 13) and/or moderate-high (n = 20) use (Table 2). Figure 3 illustrates the extent of research use in general by year of publication. A peak in the number of articles reporting general research use occurred in 1995 to 1999, and reports of high general research use starting in 2005.

**Figure 4:**
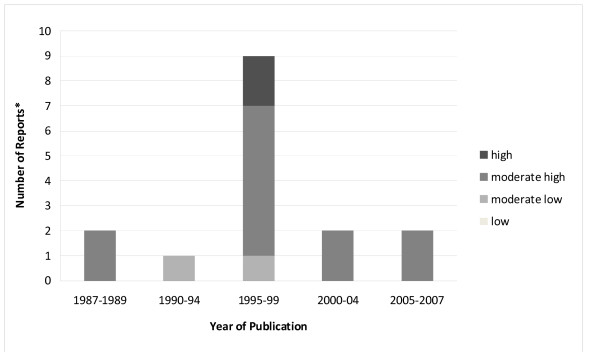
**Extent of use of specific research findings by year of publication (n = 14 articles)**. One study (Davies, 1999) reports three levels of research use dependent on practices assessed. Thus, 14 articles account for 16 measures reported in this figure.

Use of specific research findings was measured in 14 studies, including those in which the NPQ was administered [5,26,27,33,49-56], one study that used a other multi-item measure [87], and an additional study that employed a single-item measure [99]. Use of specific research ranged from moderate-low to high with the overwhelming majority of studies reporting moderate-high research use. The study that used a single-item measure [99] reported moderate-low, moderate-high or high use, dependent on the practices assessed. Figure 4 illustrates the extent of use of specific research findings by year of publication. Similar to reports of general research use, there was a peak in publications during the 1995 to 1999 year range. The only reports of high use of specific research findings occurred between 1995 and 1999.

Kinds (including instrumental, conceptual, persuasive and overall) of research use were measured in six studies [25,64,66,70,76,84], each of which employed Estabrooks' measures. Across these studies, instrumental research use was reported as moderate-high to high, conceptual and persuasive research use ranged from moderate-low to high, and overall research use ranged from moderate-high to high. The majority of publications examining kinds of research use are from 2005 onwards. There were no reports of low research use; the first reports of high research use occurred in 2003 (conceptual research use), 2005 (instrumental and overall research use), and 2007 (persuasive research use).

## Discussion

The various ways in which research use is conceptualized (*i.e.*, as a process or an outcome, as a general concept or as kinds -- instrumental, conceptual, persuasive, overall) coupled with the use of multiple instruments to assess nurses' use of research, challenges clinicians' and investigators' ability to directly compare findings from various studies to determine the extent to which nurses use research in clinical practice. In this review, by quantifying nurses' use of research as low, moderate-low, moderate-high, or high, we were able to indirectly compare the results of the 55 included articles and conclude that the extent to which nurses report using research in clinical practice is, on average, moderate-high (with 38 of the 55 articles reporting research use in the moderate-high range) (Table 2). Caution must be used when interpreting this finding, however, because we combined different instruments (and conceptualizations of research use) in reaching this conclusion.

### Specific versus general research use

An examination of the extent of research use elicited by different instruments revealed little variation in the scores regardless of whether nurses were asked to report on their use of specific research-based practices (*e.g.*, NPQ) or on their use of research generally (*e.g.*, RUQ). Most articles that used the NPQ (n = 10 of 12) were ranked in the moderate-high research use category. Of the ten articles that used the RUQ, eight were classified in the moderate-high category. Limited variation in reported research use for the NPQ and the RUQ suggests that an instrument effect may be at play. As such, a propensity towards moderately high use of research may reflect either a regression to the mean effect, or a lack of sensitivity of the instruments to detect changes in research use over time.

Anecdotally we know that nurses find it challenging to respond to general questions about the extent to which they use research in practice. Importantly, such omnibus questions require nurses to first be aware that they are using research. If nurses are using research but are not aware they are, this type of question should lead to under-reporting of research utilization. However, in this review, nurses reported, on average, moderate-high use. Instruments containing questions that relate to the use of specific research evidence, on the other hand, provide a context for respondents and enable them to relate their responses to their work. This was illustrated in a recent international study with nursing service providers in Canada and Sweden [100]. In this study, the investigators needed to provide concrete examples of research-based practices to stimulate nurses' reflection of their use of research in practice. This, in turn, however has the potential for increasing any social desirability effect that may exist.

### Different kinds of research use

Six reports [25,64,66,70,76,84] in this review assessed the extent to which nurses reported different kinds of research use. Overall, use was highest for conceptual research use, followed by instrumental and persuasive use, with two exceptions [64,76]. The first exception is from a study of research use in Canadian nursing homes; Connor [64] reported high persuasive research use (RNs mean = 6.07, LPNs mean = 5.27 on a 7-point scale), followed by instrumental and lastly conceptual research use. This may be explained however by the context (setting/work environment) in which the nurses in this study were employed. Nurses in Canadian long-term care facilities have a largely supervisory role in overseeing the practice of healthcare aides who provide the majority of direct care. Therefore, nurses in this setting are more likely to use research persuasively in order to convince direct care providers (healthcare aides) to provide research-based care.

The second exception can be seen in a study conducted by Milner and colleagues [76]. In this study, Milner found that staff nurses and advanced practice nurses (educators and managers) both reported similar patterns (high conceptual followed by instrumental and persuasive use) in the extent to which they use research in clinical practice. However, the extent of use (for all kinds of research) reported by advanced practice nurses was higher when compared to staff nurses. Similar findings were also noted by Veeramah [9] in a study of graduate nurses and midwives in the United Kingdom. Veeramah [9] found that 67% of nurses reported a high extent of research use. The majority (63%) of nurses in this study, however, occupied senior positions with varying degrees of managerial responsibilities, autonomy, and authority, which may have been responsible for the higher extent of research use reported; nurses in management roles have greater authority to use research to implement change. These findings with respect to role are consistent with past research. While Estabrooks and colleagues [38] and Meijers and colleagues [39] located too few studies investigating role to reach a conclusion on its effect on research use, they did find consistent findings in the studies they located, with nurses in leadership roles reporting higher research use compared to staff nurses.

### The state of the science when examining extent of research use

This review describes the range of measures of research use that have been used with nurses. It paints a somewhat discouraging portrait. Although the use of research evidence to underpin practice is viewed as fundamentally important, this review demonstrates several major limitations in this area of the field.

The first major limitation relates to methodological quality. Few studies examining nurses' use of research were strong (or even moderately strong) methodologically, illustrating a need for better-designed studies. Of the 55 articles included in this review, 51 reported a cross-sectional design. This design enables researchers to capture nurses' perceptions of their use of research at a single point in time. However, restricting study to cross-sectional research limits advances in the field. For example, evidence for causal inferences that can be used to develop interventions to increase nurses' use of research and consequently improve patient care is limited with cross-sectional designs. Four studies included in this review used a quasi-experimental design. All four studies measured research use pre- and post-implementation of an intervention designed to improve nurses' use of research in practice. Three of the studies implemented a control or comparison group alongside the experimental group, but reported little consideration of confounding variables, limiting the internal validity of the studies. Future studies in the field need to use more robust quasi-experimental and experimental (*e.g.*, pragmatic RCTs) designs that take into consideration, and control for, threats to internal validity.

The second major limitation relates to the measures in use; these measures have several problems. First, there is inconsistency in the measures used, including widely varying use of language. While we were able to develop a method with which to compare findings on the extent of research use by dividing research use scores into quartiles, the lack of standard language makes it difficult to compare, contrast, and evaluate findings collected with the various instruments. Second, with the exception of the NPQ, none of the research use measures identified were developed explicitly using a relevant theoretical framework. As well, none of the studies examined reported the use of measurement theory (*i.e.*, classical test score theory or item response theory [101,102]) in the design or evaluation of the instrument. Finally, all of the instruments used self-report measures of research use. The advantages of self-report are well known. Whether done using paper and pencil, online, or computer-assisted telephone or personal interview, it has the benefits of cost efficiency, convenience, and time efficiency for researchers. Despite these advantages, self-report measures are also often criticized. They are reports of 'perception' and therefore, not 'objective' measures. With respect to the measurement of research use, self-report instruments are further criticized because of an inability to clarify items and thus what is meant by 'research,' an inability to probe to more fully understand what nurses mean when they report research use (or non-use), and reduced ability by nurses to recall how often they use research. The most frequent criticisms however are that such measures offer the potential for social desirability bias [103] and rely on nurses' ability to recognize that they are using research. One way to reduce social desirability bias is to pay careful attention to instrument design (*e.g.*, attention to item wording, item order, response options, and pre-testing) [103-105]. To this end, and positively, some of the research use measures identified in this review, while reliant on self-report measurement, have undergone extensive feasibility and pre-testing [24,25,27]. Further, if social desirability bias were an issue for the studies identified, we would expect to see an increase in the extent of nurses reported research use in recent years, given the current drive towards evidence-based nursing practice, of which research use is one component. Instead, extent scores have remained relatively consistent over time into the early 2000's, when the shift towards evidence-based practice emerged. We recommend that future studies be conducted that: examine the scores obtained from the self-report research use instruments identified in this review along side other forms of assessment (*e.g.*, chart audit, think-aloud, observation), and attempt to causally associate research use scores with practice improvements and/or improved patient outcomes. Such studies will not only assess the validity of the research use scores obtained with these instruments and concerns with using self-report measurement, but also significantly advance the field. Until we have accurate and reliable measures of research use, it will not be possible to know, with any degree of certainty, whether intervention efforts are increasing nurses' use of research. Thus, in order to progress the field, robust measures of research use are critical.

Third, is conceptualization (theory) and resulting operationalization (the scales and scoring methods) of current research utilization measures. The published literature is characterized by multiple conceptualizations of research utilization. This influences how we define research utilization and, importantly, how we measure the construct and interpret the scores obtained from such measurement. Two conceptualizations dominating the field are: research utilization as a process (*i.e.*, consists of a series of stages/steps) and research utilization as a variable or discrete event (also referred to as the 'variance' approach). Both types of measures were evident in this review. Assigning meaning to the scales used, however, regardless of whether the measure follows a process or variable conceptualization, in many cases remains unclear. For example, scores (called Total Innovation Adoption Scores) ranging from 0 to 4 are theoretically possible (and have been reported) in studies using the NPQ [26] (a process measure) to assess research utilization. A stage of adoption (as per Rogers' Innovation Decision Process Theory [106,107]) is then assigned to the resulting score: 0 to 0.49 (unaware), 0.5 to 1.49 (aware), 1.5 to 2.49 (persuasion), 2.5 to 3.49 (use sometimes), and 3.5 to 4.0 (use always). Using this schematic, a research utilization score of '1' is feasible. This score is interpreted as the respondent is aware of the research findings and is 1 (on a 0 to 4 scale) with respect to 'using research.' What is unclear is how this is interpreted as 'using research' when no 'use' is actually occurring? While no one would disagree that awareness is desirable and in many cases, necessary, for research utilization to occur, awareness is not 'research use' *per se *nor does it guarantee that research use will occur. In line with Rogers' Diffusion of Innovations theory (from which this scoring is stated to have been developed), an individual may be aware of the innovation (research findings) and still choose not to use it in practice if they are not persuaded of its effectiveness. This scoring method gives the impression research use is occurring when it is not, painting a more optimistic picture of research use than actually exists. Similar pictures are painted with variable measures of research utilization. For example, items in the RUQ [63] are scored on a 5-point Likert agreement scale (1 - strongly disagree, 2 - disagree, 3 - neither agree nor disagree, 4 - agree, 5 - strongly agree). But it is unclear how to interpret these scores as quantitative measures of research use. For instance, an overall scale score of '2' is interpreted as the nurse is just below average with respect to their 'use' of research. However, a score of '2,' according to the scale descriptors, would imply the same nurse 'disagreed' with most of the statements about their use of research. As with the NPQ, we contend that these scores also paint a more optimistic picture than actually exists. Similar scaling issues can be found in the remaining research utilization measures. As a result of these scoring problems, we believe that the extent to which nurses use research in their practice that is portrayed in the literature (and by association, in our synthesis) is higher than what actually exits.

Fourth, although nurses' research use has been measured by various instruments and has been studied for nearly 40 years, no benchmarking has been done. We thus have no 'gold standard' against which to compare the findings of any studies measuring nurses' use of research. A standard measure or set of measures of research use would help in such an effort. Equally, if not more important, is work that enables researchers and decision makers to evaluate the effect of different levels of research use on patient outcomes.

Progress in this field depends on having robust measures of research use. Fundamental to achieving this, we believe, are: an understanding of the validity and reliability of the measures that have been used to date; instrument development work that focuses on strengthening measurement accuracy; development of benchmarks for research use; and investigating the impact of varying levels of research use on patient outcomes. To date, there has been little emphasis on examination of the effects of varying levels of research use on patient and other outcomes (*e.g.*, system, provider). Despite a strongly held assumption that integrating research evidence into practice will improve patient outcomes, none of the 55 articles included in this review examined associations between research use and patient or provider outcomes. Therefore, we could not assess the effect of research use on patient outcomes.

### Limitations

A limitation of our work is that we deliberately excluded the terms 'evidence-based practice' and 'decision making' from our search. Our rationale for doing so was that evidence-based practice refers to the application of a range of sources of evidence, including patient preference, the clinician's expertise, and resources, in addition to research evidence [108]. Decision making also takes into account multiple information-seeking processes and resources. Research use, on the other hand, refers very specifically to the use of research in practice. We acknowledge that by excluding studies that measured evidence-based practice and/or decision making, we may have failed to capture the role of research use revealed in such studies. Further, our finding of moderate-high use may not hold if these additional studies were included. Future research should examine the extent to which nurses use other information sources, in addition to and in combination with research, to make clinical decisions.

## Conclusion

From this review we are able to conclude that the extent to which nurses report research use in their daily practice is, on average, moderate-high, and has remained fairly consistent over time into the early 2000's, when increasing awareness of the evidence-based practice movement may have influenced the number of reports published on research use alone. Our finding suggests a more optimistic picture than we believe exists. Our combined nursing experience, which exceeds eight decades, causes us to question the accuracy of this finding. In an attempt to understand this finding, we turn to other plausible explanations. It is possible that the method we used to calculate the extent of research use was not sensitive enough to detect changes in research use over time and thus provided an overly positive result. Assuming, however, that our method was sufficiently sensitive, other plausible explanations for the finding include: lack of construct validity evidence of the research use scores obtained; scaling and other instrument concerns; self-report measurement biases (*i.e.*, recall and social desirability); and low methodological quality of the studies included in the review. The results of this review, coupled with our hesitancy in accepting the finding that nurses' use of research is moderate-high, suggest a need for advances in the field, starting with a systematic and detailed assessment of the validity of current research use instruments used with nurses; more robust research designs including longitudinal research programs and programmatic research that examines nurses' research use and its link to patient and system outcomes; and the establishment of a benchmark against which to compare findings of studies measuring nurses' use of research.

## Competing interests

The authors declare that they have no competing interests.

## Authors' contributions

JES designed the review; developed the search strategy; undertook the article selection; participated in data extraction, quality assessment, and data synthesis; and made major contributions to drafting the manuscript. AMH, AMB, and HMO participated in data extraction and quality assessment, data synthesis, and contributed to drafting the manuscript. SJC participated in data synthesis and drafting the manuscript. CAE provided guidance throughout the study and critical commentary on manuscript drafts. All authors provided commentary on and approved the final manuscript.

## Supplementary Material

Additional file 1 **Characteristics of articles using the NPQ to assess research use**. A summary of data extraction and extent calculation on studies that used the NPQ. Click here for file

Additional file 2 **Characteristics of articles using the RUQ to assess research use**. A summary of data extraction and extent calculation on studies that used the RUQ. Click here for file

Additional file 3 **Characteristics of articles using other multi-item measures to assess research use**. A summary of data extraction and extent calculation on studies that used other multi-item instruments. Click here for file

Additional file 4 **Characteristics of articles using single-item measures of research use**. A summary of data extraction and extent calculation on studies that used single-item measures of research utilization. Click here for file

Additional file 5 **Excluded Articles based on Full-Text (n = 78)**. Citation and reason for exclusion for 78 articles retrieved but not included in the review. Click here for file

Additional file 6 **Quality Assessment**. A summary of quality assessment of the 55 articles included in the review. Click here for file
