# Supplementary material for: To what extent do nurses use research in clinical practice? A systematic review
Source: Implement Sci. 2011 Mar 17;6:21. doi: 10.1186/1748-5908-6-21 (PMC3068972; doi:10.1186/1748-5908-6-21)
Supplement: Additional file 1 — Characteristics of articles using the NPQ to assess research use. A summary of data extraction and extent calculation on studies that used the NPQ. [file 1748-5908-6-21-S1.DOC]

**Additional file 1. Characteristics of articles using the NPQ to assess research use**

| **Citation** | **Country** | **Setting** | **Sample** | **Reliability**  **&**  **Validity** | **Adoption by Practice** | **Adoption by Nurse** | **Mean Adoption Score Stage** | **Extent^1^** | **Quality** |
| --- | --- | --- | --- | --- | --- | --- | --- | --- | --- |
| Brett, 1987  Brett, 1989 | United States | 19 acute care hospitals | Subjects**:** Registered nurses  Characteristics:  - Associate degrees or diplomas (51%); baccalaureate (40%)  - Staff nurses (81%)  Size: N = 216  Response rate: 63% | Reliability:  Test-retest (pilot):  r = 0.83  α (NPQ)= 0.95  α (subscales) = 0.68 to 0.95  Validity Content by appropriateness of the nursing practices | Of 14 practices  Aware only: 14%  Persuasion: 50%  Use sometimes: 29%  Use always: 7% | Aware: 34% to 95%  Persuaded: 28% to 92%  Using: 31% to 93% | 2.17  ‘Persuasion’ | Moderate-High | Moderate-High |
| Coyle, 1990 | United States | Acute care hospital | Subjects: Registered nurses, registered practical nurses  Characteristics**:**  - Associate degrees or diplomas (61%), baccalaureate (33%)  - Staff nurses (73%)  Size: N = 113  Response rate: 56% | Reliability:  α (NPQ) = 0.91  α (subscales) = 0.79 to 0.90  Validity: Content by appropriateness of the nursing practices | Of 14 practices  Aware only: 36%  Persuasion: 36%  Use sometimes: 21%  Use always: 7% | Aware: 25% to 83%  Persuaded: 7% to 91%  Using: 23% to 80% | 1.96  ‘Persuasion’ | Moderate-Low | Moderate-High |
| Barta, 1995 | United States | 409 baccalaureate degree nursing programs | Subjects: Pediatric nurse educators  Characteristics:  - Masters (94%)  - Assistant professors (49%) and associate professors (29%)  Size: N = 213  Response rate: 52% | Reliability: α = 0.74  Validity: Content by expert panel of three paediatric nurses | Of 8 practices  Aware only: 0%  Persuasion: 13%  Use sometimes: 75%  Include always: 13% | Not Reported | 2.98  ‘Use sometimes’ | Moderate-High | Moderate-High |
| Michel, 1995 | United States | Members of an university-associated STTI Honor Society | Subjects: Nurses  Characteristics:  - Baccalaureate (45%), masters (49%)  - Mean yrs in nursing = 14.5  57% reported their practice area as clinical (9%) education, 14% admin., 20% other)  Size: N = 167  Response rate: 84% | Reliability  α(NPQ) = 0.85  α(subscales) = 0.73 to 0.84  Validity: Research findings derived from published literature | Of 5 practices  Aware only: 20%  Persuasion: 60%  Use sometimes: 20%  Use always: 0% | Not Reported | 2.21  ‘Persuasion’ | Moderate-High | Moderate-High |
| Berggren, 1996 | Sweden | Members of a county division of the Swedish Midwives’ Association | Subjects: Midwives  Characteristics:  - Degree (32%), other specialist nurse education (8%)  - Mean yrs in midwifery = 11.2  - Staff midwives (89%)  Size: N = 108 (returned)  Response rate: 74% | Reliability  α(MPQ) = 0.68  α(subscales) = 0.59 to 0.76  Validity: Midwifery practices taken from doctoral dissertations  and articles published  in the journal of the Swedish Midwives’ Association | Of 14 practices  Unaware: 7%  Aware only: 21%  Persuaded: 43%  Use sometimes: 14%  Use always: 14% | Aware: 17% to 100%  Persuaded: 14% to 100%  Using: 2% to 87% | 2.06  ‘Persuasion’ | Moderate-High | Moderate-Low |
| Rutledge, 1996 | United States | Oncology settings (hospitals, outpatient clinic, hospice, home care, etc.) | Subjects: Staff nurses  Characteristics:  - Associate degree/ diploma (58%), bachelor’s degrees (36%), master’s degree (7%)  - Mean age = 40 yrs  - Mean yrs in oncology = 7.4  - Employed in hospitals (59%)  Size: N=1100  Response rate: 39% | Reliability: α = 0.75  Validity: Content by expert panel | Of eight practices:  Use sometimes: 88%  Use always: 13 % | Aware: 53% to 96%  Persuaded: 67% to 97%  Using: 18% to 70% | 3.33  Use sometimes  *Of only those aware* | High | Moderate-Low |
| Thompson, 1997 | United States | Acute care hospitals | Subjects: Registered nurses  Characteristics:  - Baccalaureate (52%)  - Mean age = 39.5 yrs  -Mean yrs in nursing = 15.9  Size: N = 212  Response rate: 42% | Reliability: α(Pilot) = 0.74  Validity: Content by expert panel; CVI = 0.94 | Of 14 practices  Aware only: 7%  Persuasion: 0%  Use sometimes: 50%  Use always: 29% | Aware: 28% to 99%  Persuaded: 27% to 92%  Using: 36% to 99% | 2.88  ‘Persuasion’ | Moderate-High | Moderate-High |
| Rodgers, 2000, A study  Rodgers, 2000,  The extent | United Kingdom (Scotland) | 25 hospitals (medical and surgical wards) | Subjects: Registered nurses  Characteristics:  -Registration only (64%), professional studies/ first degree (5%)  - Majority yrs in nursing = 5 to 10 (25%)  -Staff nurses (69%)  Size: N = 680  Response rate: 73% | Reliability: α = 0.63  Validity: Content by expert panel of nurse researchers and educators | Of 14 practices  Aware only: 7%  Persuaded: 36%  Use sometimes: 36%  Use always: 21% | Unaware: 3% to 61%  Aware only: <1% to 7%  Persuaded only: 1% to 28%  Using: 8% to 85% | 2.65  ‘Persuasion’ | Moderate-High | Moderate-High |
| Squires, 2007 | Canada | Adult acute care hospital | Subjects: Registered nurses  Characteristics:  - Diploma (40.3%), diploma + specialty course (22.2%), baccalaureate (37.1%), masters or higher (0.4%)  - Mean yrs in nursing = 11.4 years  - Medical (23.4%), surgical (22.2%), and combination units (18.5%), critical care (35.9%)  Size: N = 248  Response rate: 53.5% | Reliability: α = 0.82  Validity: Research-based practices were identified from research literature | Of eight practices  Aware: 25%  Persuaded: 25%  Use sometimes: 50% | Aware:13% to 89%  Persuaded: 23% to 87%  Any use: 8% to 89% | 2.27  ‘Persuasion’ | Moderate-High | Moderate-High |
| Carlson, 2006 | United States | Two hospitals (oncology units) | Subjects**:** Registered nurses  Characteristics  - Associate degree in nursing (55%)  - Mean age = 39.71 yrs  - Mean yrs in nursing =13.19  Size: N = 443  Response rate: 46.9% | Reliability: α = 0.78  Validity: Content by expert panel | Of three practices  Aware: 8%  Persuaded: 20%  Use sometimes: 44%  Use always: 25% | Aware: 85% to 88%  Persuaded: 79% to 84%  Any use: 58% -79% | 2.77  ‘Use sometimes’ | Moderate-High | Moderate-Low |

**^1^= TIAB score range 0-4. Extent calculated by dividing TIAB score range into 4 equal quartiles as follows: low (0-0.99), moderate-low (1.00-1.99), moderate-high (2.00-2.99), high (3.00-4.00)**
