# Supplementary material for: To what extent do nurses use research in clinical practice? A systematic review
Source: Implement Sci. 2011 Mar 17;6:21. doi: 10.1186/1748-5908-6-21 (PMC3068972; doi:10.1186/1748-5908-6-21)
Supplement: Additional file 2 — Characteristics of articles using the RUQ to assess research use. A summary of data extraction and extent calculation on studies that used the RUQ. [file 1748-5908-6-21-S2.DOC]

**Additional file 2. Characteristics of articles using the RUQ to assess research use**

| **Citation** | **Country** | **Setting** | **Sample** | **Reliability & Validity** | **Mean Research Utilization Score** | **Extent^1^** | **Implement Specific Research Findings into Practice Item** | **Quality** |
| --- | --- | --- | --- | --- | --- | --- | --- | --- |
| Champion, 1989 | United States | Community hospital | Subjects**:** Registered nurses  Characteristics  - Baccalaureate degree (54%), masters or higher degree (16%)  - Mean age = 36 yrs  -Mean yrs experience in nursing = 11.28    Size: N = 59  Response rate: 39% | Reliability  α (use subscale) = 0.92  α (sub-scales) = 0. 84 to 0.94  Validity: Content by expert panel | 3.48 | Moderate-High | N/A | Weak |
| Lacey, 1994 | United Kingdom | Acute care hospital | Subjects**:** Registered nurses  Characteristics:  Nurses working at F/G level  Size:N = 20  Response rate: N/A | Reliability: Not reported  Validity: Validity of  self-reports assessed by follow-up interviews | 3.79 | Moderate-High | N/A | Weak |
| Prin 1997 | United States | Medical-surgical units in a large, university medical center | Subjects**:**  Female clinical nurses  Characteristics:  Mean age = 34.3 yrs  Size: N = 121  Response rate: 40% | 9 of the 10 items developed by Champion & Leach were used  Reliability: α = 0.94  Pilot testing indicated one item contributed to low reliability. Scale range went from 9 to 45 with deletion of this item.  Validity: Content by three  nursing informatics experts | 30.43  (SD 7.10)  Mean on 5-point scale = 3.38 | Moderate-High | N/A | Moderate-Low |
| Hatcher, 1997 | Canada | Acute care hospital | Subjects**:** Registered nurses, registered practical nurses  Characteristics:  - Diploma (72%), baccalaureate (20%), graduate degree (7%)  - Mean yrs in nursing = 15.75  -Registered practical nurses (7.5%), registered staff nurses (70%), unit managers (9%), educators (7.5%), administrators (6%)  Size: N=174  Response rate: 44% | Reliability: Refer to Champion and Leach 1989  Validity: Not reported | 3.42 | Moderate-High | N/A | Moderate-Low |
| Hansen, 1999 | United States | Large medical center | Subjects**:** Emergency room nurses  Characteristics:  - Baccalaureate degree (43.8%), associate degree (37.3%), diploma (18.8%), certified in a nursing speciality (43%)  - Mean age = 39.2 yrs  Size: N = 64  Response rate: 56% | Reliability: α (research utilization scales) = 0.79 to 0.93  Validity: Predictive (previous studies)-RU as dependent variable best predicted  by research attitude and availability | 3.25 (SD 0.65) | Moderate-High | N/A | Weak |
| Humphris, 1999 | United Kingdom | Acute care trusts | Subjects**:** Registered Nurses (RN), Diabetic Nurse Specialists (DNS)  Characteristics  Reported for DNS  -Baccalaureate (17.7%), masters (4%), doctorate (0.7%)  -Mean yrs experience in specialist practice = 7.1  Size: DNS N = 299  Response rate DNS = 72% | Reliability: Not reported  Validity: Not reported | Not Reported | Moderate-High (based on single item %) | DNS: 74% | Moderate-Low |
| Tranmer, 2002 | Canada | Acute care hospital | Subjects**:** Registered Nurses  -high, low, and controlled exposure to research  Characteristics:  Three groups  High/low/control  **-**Age (30 to 39 yrs (54%/48%/40%)  -Highest level of education  Diploma (81%/77%/89%)  Baccalaureate (11%/14%/9%)  Masters (0%/0%/0%)  -Yrs worked (0.02)  (8 ± 5 / 12 ± 5 / 8 ± 5)  Size: N = 190  Response rate: 39% (baseline) ; 37% (one year later) | Reliability: α = 0.93  Validity: Not reported | Baseline:  High: 3.40* (SD 0.52)  Low: 2.98 (SD 0.70)  Control: 3.07 (SD 0.69)  Post-Intervention:  High: 3.46 (SD 0.71)  Low: 3.03 (SD 0.68)  Control: 3.20* (SD 0.69)  * = significant difference between groups (p<0.05) | Moderate-High | N/A | Moderate-High |
| Wallin 2003 | Sweden | Acute care, psychiatry, primary care, and nursing homes | Subjects**:** Registered nurses who participated in a quality improvement (QI) training course (86 staff nurses, 33 nurse managers)  Characteristics:  Group 1 (continued QI) vs. 2 (discontinued QI) (ns)  -Mean yrs in nursing = 22.0 vs. 19.8  -Nurses/nurse managers 31/15 vs. 54/18  Size: N = 119  Response rate: 70% | Reliability: Not reported  Validity: Not reported | Group 1 -  2.67 (SD 0.87)  Needed to be reverse coded ( so mean = 3.33)  Group 2 -  2.94 (SD 0.75)  p< 0.10  Needed to be reverse coded ( so mean = 3.06) | Moderate-High | Group 1 to 46%  Group 2 to 16% | Moderate-Low |
| McCloskey 2005 | United States | 5 hospitals | Subjects**:** Registered nurses  Characteristics:  -Masters (19.6%), baccalaureate (48.5%), associate degree/ diploma (31.5%)  - Mean age = 43.9yrs  - Mean yrs in nursing = 17  Size: N = 270  Response rate: 9.6% | Reliability: α = 0.93  Validity: Content by panel of experts | 2.96 | Moderate-Low | N/A | Weak |
| Nash 2005 | United States | Idaho | Subjects**:** Registered Nurses  Characteristics:  -AND (35%), diploma (5%), baccalaureate (44%), masters (14%)  Size: N = 82  Response rate: 33% | Reliability: α = 0.91  Validity: Refer to Champion and Leach 1989 | 2.9 (SD 0.55) | Moderate-Low | N/A | Weak |

**^1^Reserach utilization Subscale range of 1 to 5**. **Extent calculated by dividing mean subscale score range into 4 equal quartiles as follows: low (1.00 to 1.99), moderate-low (2.00 to 2.99), moderate-high (3.00 to 3.99), high (4.00 to 5.00)**
