# Supplementary material for: To what extent do nurses use research in clinical practice? A systematic review
Source: Implement Sci. 2011 Mar 17;6:21. doi: 10.1186/1748-5908-6-21 (PMC3068972; doi:10.1186/1748-5908-6-21)
Supplement: Additional file 3 — Characteristics of articles using other multi-item measures to assess research use. A summary of data extraction and extent calculation on studies that used other multi-item instruments. [file 1748-5908-6-21-S3.DOC]

Additional file 3. Characteristics of articles using other multi-item measures to assess research use

| **Citation** | **Country** | **Setting** | **Sample** | **Reliability**  **&**  **Validity** | **Instrument** | **Research Use** | **Extent^1^** | **Quality** |
| --- | --- | --- | --- | --- | --- | --- | --- | --- |
| Pelz, 1981 | United States | Medical/  surgical nursing units | Subjects: Registered nurses- upper level roles (non-innovation team)    Characteristics :  Not described  Size:  **Hospital:**  (Experimental, Comparative)  Year 1 (pre-test): N = 15, 15 Year 2: N = 13, 15 Year 3: N = 6, 9  **Nurses:** Range 4 to 32 per hospital  Response rate:  **Hospital RR:** Of 17 experimental hospitals, two were used as pilot sites (results not reported) and two dropped out during the first year.  **Nurse RR:** Mean 69%  (58% to 81% from each site) | Reliability: α = 0.87  Validity: Not reported | Questionnaire with around 430 items. One direct RU index consisting of five items to directly measure research use.  Scored 0 to 5 times in the past year    a) Reviewed research literature in an effort to identify new knowledge for use in your practice.  b) Evaluated a research study to determine its value for practice.  c) Transferred knowledge included in the results of the research studies into useful practice activities  d) Planned for the implementation and evaluation of new research-based practices  e) Discontinued or rejected a practice activity because of knowledge included in the results of research studies  . | Experimental, Comparative: (NS)  Year 1: 1.32, 1.29  Year 2: 1.38, 1.26  Year 3: 1.56, 1.15 | Moderate-Low | Weak |
| Varcoe, 1995 | Canada | Acute care hospital (Medical surgical and critical care) | Subjects**:** Registered nurses  Characteristics  - Diploma (45%), baccalaureate (54%)  - 56% graduated less than 10 years prior  - Mean age baccalaureate 34.5 yrs /diploma 38 years  Size: N = 183  Response rate: 42% | Reliability  α(General RU) = 0.87  α(Specific findings) = 0.87  Validity: Content-pilot testing  and peer review | The Research Use in Nursing Practice Instrument (Alcock 1990). 10 general statements on general research use and 10 on use of specific research practices (from Brett 1987)  General use: 4-pt scale: not at all (1), sometimes (2), frequently (3), always (4)  Use of specific findings: 3-pt scale: never (1), sometimes (2), always (3) | General Research Use  Mean: 22.7 (SD 4.91) (22.7/10 = 2.25)  Range: 10 to 38; All statements rated as sometimes used by at least 80% of nurses  Specific Findings:  Mean: 2.15 (SD 0.36)  (based on seven practices)  Three practices rated as N/A by over 30%; these excluded from analysis  -With one exception (catheter clamping) each finding was rated as ‘use sometimes’ by >50% of nurses | Moderate-Low (general research use)  Moderate –High (use of specific practices) | Moderate-Low |
| Stiefel,1996 | United States | Two hospitals (20 nursing units) | Subjects**:** clinical nurses from adult medical, oncology, surgical, and critical care  Characteristics:  AND (23%), diploma (11%), baccalaureate (63%), masters (3%)  - Mean age = 33.8 yrs  - Mean yrs in nursing = 8.76  Size: N = 100  Response rate: Not reported | Reliability:  Test-retest (pilot) r = 0.876  α (pilot) = 0.94 to 0.95  Factor 1: α = 0.92  Factor 2: α = 0.89  Factor 3: α = 0.93  Validity: Content by four NRU experts  Factor analysis  Factor 1: 0.56 to 0.88  Factor 2: 0.43 to 0.74  Factor 3: 0.57 to 0.65 | Nursing Research  Utilization Survey  (NRUS) (developed and tested by the researcher for this study)  18 items measuring respondent’s reported participation in research utilization activities  5-pt Likert scale: never-always  Scoring range: 18 to 90 | Mean: 54.12 (SD 11.96)  Mean: (5-point scale) = 3.00  Range: 24.00 to 83.00 | Moderate –High | Weak |
| McCleary 2002  Use of the EROS…  Report 1/2 | Canada | Paediatric teaching hospital | Subjects**:** Registered nurses  Characteristics:  - Mean age 40.5 yrs  - Mean yrs in nursing 18.1  Size: N = 185  Response rate : 35% | Reliability: α = 0.87  Validity: Content (Pain *et al*.  1996): items constructed using data from two focus groups of clinicians, researchers, and managers; pilot tested and revised based on feedback | Edmonton Research Orientation Survey (EROS) containing an evidence-based practice subscale.  5-pt Likert scale: strongly disagree (1) to strongly agree (5) | Evidence-based practice:  Mean: 3.56 (SD 0.58)  Range: 1.9 to 5.0 | Moderate –High | Moderate-Low |
| McCleary, 2003  Association between nurses’ education…  Report 2/2 | Canada | Paediatric acute care hospital | Subjects**:** Registered nurses  Characteristics  - Baccalaureate (43.4%), masters (4%)  Size: N = 175  Response rate : 33.3% | Reliability: Refer to Pain 1996  Validity: Refer to Pain 1996 | Edmonton Research Orientation Survey (EROS) with one evidence-based practice subscale.  5-pt Likert scale: strongly disagree (1) - strongly agree (5) | Mean: 3.57 (SD 0.59) | Moderate –High | Weak |

**^1^Extent Calculations**

- **Peltz, 1981. 0 to 5 scale range. Extent calculated by dividing mean scale score range into 4 equal quartiles as follows: low (0 to 1.24), moderate-low (1.25 to 2.49), moderate-high (2.50 to 3.74), high (3.75 to 5.00)**
- **Varcoe, 1995. Specific practices 1 to 3 scale range. Extent calculated by dividing mean scale score range into 4 equal quartiles as follows: low (1.00 to 1.49), moderate-low (1.50 to 1.99), moderate-high (2.00 to 2.49), high (2.50 to 3.00). General research use, 1 to 4 scale range. Extent calculated by dividing mean scale score range into 4 equal quartiles as follows: low (1.00 to 1.74), moderate-low (1.75 to 2.49), moderate-high (2.50 to 3.24), high (3.25 to 4.00)**
- **Stiefel, 1996; McCleary 2002; McCleary 2003. 1 to 5 scale range. Extent calculated by dividing mean subscale score range into 4 equal quartiles as follows: low (1.00 to 1.99), moderate-low (2.00 to 2.99), moderate-high (3.00 to 3.99), high (4.00 to 5.00)**
