# Supplementary material for: To what extent do nurses use research in clinical practice? A systematic review
Source: Implement Sci. 2011 Mar 17;6:21. doi: 10.1186/1748-5908-6-21 (PMC3068972; doi:10.1186/1748-5908-6-21)
Supplement: Additional file 4 — Characteristics of articles using single-item measures of research use. A summary of data extraction and extent calculation on studies that used single-item measures of research utilization. [file 1748-5908-6-21-S4.DOC]

**Additional File 4. Characteristics of articles using single item measures of research use**

| **Citation** | | **Country** | **Setting** | **Sample** | **Reliability**  **and**  **Validity** | **Findings** | **Extent^1^** | **Quality** |
| --- | --- | --- | --- | --- | --- | --- | --- | --- |
|  | Past, Present, Future | | | | | | | |
| Bostrom, 1993 | | United States | 12 health care agencies | Subjects**:** Registered nurses    Characteristics  -Baccalaureate (50%), graduate (15%), working towards higher degrees (15%)  -10 yrs or less in nursing (50%)  Size: N = 1588  Response rate: 23% | Reliability:  α (Bostrom *et al.* 1991, overall) = 0.88  Validity: Previously reported (Rizutto *et al.* 1990, unpublished) | Made a research-based practice change  - **Present (most recent six months): 15.9%**  - Past (>6 months ago): 23.4%  Use of research findings in clinical practice: Not reported | Low (current use) | Weak |
| Rizzuto, 1994 | | United States | 9 California health care agencies | Subjects**:** Nurses  Characteristics:  -Baccalaureate (52%), masters (15%), working towards higher degree (15%)  -Age 30 to 39 yrs (42%)  -Mean yrs employed = 12.7  -Staff nurses (80%)  Size: N = 1217  Response rate: 29% | Reliability  α (3 use subscales):  Past = 0.89  Present = 0.86  Future = 0.96  Validity: Face by panel of nurse researchers who were employed in the clinical setting | Using research findings as a basis for changing nursing practice  - Past (>6 months ago): 24.6%  - **Present (most recent 6 months): 15.9%**  - Future (within next year): 42.5% | Low (current use) | Weak |
| Butler, 1995 | | Canada | Large tertiary hospital | Subjects**:** Nurses  Characteristics: (leadership/staff)  -Mean age = 41 yrs/35 yrs  -Mean yrs in nursing = 20/13  -Baccalaureate (47%/17%), diploma (30%/72%)  Size:  N = 38 from leadership group – head nurses and administrative group supervisors  N = 328 from staff nurse group  Response rate: 61% | Reliability: Not reported for the questions related to research involvement  Validity: Not Reported | Use findings in practice  Leadership Group (n = 38)  Previous: 89.5%  Present: 71.0%  Future: 97.4%  Staff Nurses (n = 310)  Previous: 52.6%  **Present: 30.3%**  Future: 77.4% | Moderate- Low (current use: staff nurses)  Moderate-High (current use: leadership nurses) | Weak |
| Brown, 1997 | | United States | 29 health care facility locations | Subjects**:** Registered nurses  Characteristics  -Baccalaureate (48%), masters or PhD (24%)  -Mean yrs in nursing = 19  -Hospital-based (59%), clinic based (39%)  -Staff nurse (50%), supervisory/managerial/educator (37%), clinical experts (13%)  Size: N = 753    Response rate: 58% | Reliability: Not reported  Validity: Not reported | **Used research findings for a particular patient’s care (past year): 71%**  **Used research findings to change practice (past year): 66%**  Apply research findings in practice (future intent): 86% | Moderate- High (current use) | Moderate - Low |
|  | Parahoo Measure | | | | | | | |
| Parahoo, 1998  Report 1 of 5 | | United Kingdom | 23 hospitals in 14 Trusts | Subjects: Nurses  Characteristics:  -Baccalaureate (9%), post-registration diploma (11%)  -Staff nurses (71%), charge nurses (10.5%), enrolled nurses (5.3%), specialist nurses (3.5%), managers (1.1%)  -Psychiatry (20%), surgical (19.7%), medical (15.4%), learning disability (6.4%), theatres (5.6%), elderly care (4.3%), intensive care (3.6%), gynecology and obstetrics (3.1%), paediatrics (2.8%), accident and emergency (2.4%), outpatients (1.1%), other (15.9%)  Size: N = 1368  Response rate: 52.6% | Reliability: Not reportedValidity: Content by panel of three experts | Use of research in clinical practice  Never: 5.5%  Seldom: 5.4%  Sometimes: 53.3%  Frequently: 26.8%  All the time: 6.1%  No response: 2.9%  Implemented specific research findings in practice within the last two years: 38.7% | Moderate-High  Moderate-Low | Moderate - Low |
| Parahoo, 1999,  A comparison  Report 2 of 5 | | United Kingdom | 14 acute care trusts (hospitals) | Subjects: All grades of hospital nurses  Characteristics:  -Staff nurses (71%), sisters/charge nurses (11%), enrolled nurses (5%) psychiatric (20%), surgical (20%), medical (15%), learning disability (6%)  Size: N = 1368  Response rate: 52.6% | Reliability: Not reportedValidity: Content by expert panel and pilot study | Use of research in clinical practice  **Pre-project 2000 (i.e., traditional training):**  Sometimes: 50%  All the time: Not reported  Low Use (i.e., never/seldom): 11.2%  High Use (i.e., freq/ all the time): 32.2%  **Post-project 2000:**  Never: 1.9%  Seldom: 5.8%  Sometimes: 61.7%  Frequently: 25.3%%  All the time: 4.5% %  Low Use (i.e., never/seldom): 7.7%  High Use (i.e., freq/ all the time): 29.8%  Implementation of new research findings in one’s own practice in the last two years- Not reported | Moderate- Low | Moderate - Low |
| Parahoo, 1999,  RU and attitudes  Report 3 of 5 | | United Kingdom | 6 main psychiatric hospitals and the psychiatric wards of six general hospitals | Subjects: Psychiatric Nurses  Characteristics:  -Staff nurses (74%), enrolled nurses (11%), ‘specialist nurses’ (6%)  Size: N = 236  Response rate: Not reported for this subset | Reliability: Not reportedValidity: Content by panel of three experts | Use of research in clinical practice  Never: 13.1%  Seldom: 9.7%  Sometimes: 52.1%  Frequently: 16.9%  All the time: 4.7%  No response: 3.4%  Implementation of specific findings in one’s own practice in the last two years: 22.5% | Moderate-High | Weak |
| Parahoo, 2000  Report 4 of 5 | | United Kingdom | 23 hospitals in 14 Trusts | Subjects: learning disability nurses Characteristics: -Completed Project 2000 diploma in learning disabilities nursing (20%)  -Grade distributions were C(2.3%), D(44.8%), E(28.7%), F(8.0%), G(14.9%), H (1.1%)  Size: N = 87  Response rate: Not reported for this subset | Reliability: Not reportedValidity: Content by three experts in the field; pilot study with 20 nurses | Use of research in clinical practice  Never: 12.6%  Seldom: 6.9%  Sometimes: 50.6%  Frequently: 21.8%  All the time: 3.4%  No response: 4.6%  Implementation of specific findings in one’s own practice in the last two years <30% | Moderate- Low | Moderate - Low |
| Parahoo, 2001  Report 5 of 5 | | United Kingdom | Hospitals in 14 acute care trusts | Subjects:  Medical and surgical nurses  Characteristics:  (Medical/surgical)  Enrolled nurse (1.9%/4.5%), RGN (60.5%/63.9%), DipHE (15.2 %/10.4%), RGN + Other (9.5%/5.6%)  Size:  Medical nurses N = 210  Surgical nurses N = 269  Response rate: 52.6% | Reliability: Not reportedValidity: Content by expert panel and pilot study | Use of research in clinical practice (ns)  **Medical Nurses:**  Never/seldom use: 7.4%  Sometimes use: 55.0%  Frequent use: 37.8%  **Surgical Nurses:**  Never/seldom use: 9.9%  Sometimes use: 60.1%  Frequent/all the time use: 29.5%  Implementation of specific findings in one’s own practice in the last two years (ns)  **Medical Nurses:** 41%  **Surgical Nurses:** 1.6% | Moderate-High | Moderate - Low |
| Valizadeh, 2003 | | Iran | 12 teaching hospitals | Subjects: Registered Nurses  Characteristics:  -Baccalaureate (86.2%), masters (5.9%), the rest were ‘technical’ nurses  -61.5% completed nursing qualifications in previous decade  -General wards (46%)  Size: N = 304  Response rate: 100% | Reliability: Not reported for RU item  Validity: Content by expert panel and pilot study by Parahoo 1999 | Use of research in clinical practice in the last two years  Never use: 29.4%  Sometimes use: 54.7%  Frequent/all the time use-15.9%  Adapted from Parahoo 1999 | Moderate- Low | Strong |
| Veeramah, 2004 | | England | Various clinical areas | Subjects: Nursing and Midwifery graduates in one health-related undergraduate programme  Characteristics:  -Baccalaureate (100%)  Senior positions (i. e. clinical grade G, H, or I) (63%)  -Clinical grades D-I (according to seniority and level of responsibility) are also reported separately  Size: N = 184  Response rate: 51.1% | Reliability: Not reported for RU measure  Validity: Content by pilot test with 12 graduates and  expert panel review by five nurses or midwifery teachers  Developed based on a review of the literature on attitudes towards research, RU in practice, and barriers to research implementation | Use of research findings to inform practice  All the time: 16.8%  Frequently: 50.5%  Sometimes: 32.6%  Never: none  Adopted from Parahoo 1998 | Moderate- High | Moderate - Low |
|  | Estabrooks’ Kinds of Research Use | | | | | | | |
| Estabrooks, 1999 | | Canada | Alberta | Subjects: Registered nurses-direct patient care  Characteristics:  -Diploma (71%), baccalaureate (25%), other (3%), missing (1%)  -Mean age = 41.7 yrs  -General hospital (42%), critical care/specialty (21%), geriatric LTC (18%), public health (9%), home care (6%), other (4%)  Size: N = 600  Response rate: 40% | Reliability: Not reported  Validity: Content by two researchers with expertise in the field | Research utilization in the past year mean scores  Overall: 4.16 to 4.71  Direct : 4.36  Indirect: 5.20  Persuasive: 3.60  7-point response alternatives  1 = never  2 = on one or two shifts  3 = unlabelled  4 = unlabelled  5 = on about half of the shifts  6 = unlabelled  7 = nearly every shift  8 = do not know | Moderate- Low (PRU)  Moderate High (IRU, CRU, ORU) | Moderate - High |
| Profetto-McGrath 2003 | | Canada | Two acute surgical units and five pediatric units in four tertiary care hospitals | Subjects: Registered Nurses  Characteristics:  -Diploma (50.9%), baccalaureate (38.9%), RNA/LPN (6.5%), masters degree (3.7%)  -Mean age = 38.0 yrs  -Mean yrs in nursing = 13.23  Size: N = 141  Response rate: Not reported | Reliability: Reported elsewhere (Estabrooks 1999a, 1999b)  Validity: Content by experts in the field; Pre-tested and pilot tested. | Mean research utilization scores  Overall: 5.4 (SD 0.60)  Conceptual: 5.8 (SD 1.5)  Symbolic: 4.8 (SD 1.8)  Instrumental: not reported.  Refer to Estabrooks, 1997  7-point response alternatives  1 = never  2 = on one or two shifts  3 = unlabelled  4 = unlabelled  5 = on about half of the shifts  6 = unlabelled  7 = nearly every shift  8 = do not know | Moderate- High (PRU, ORU)  High (CRU) | Moderate - Low |
| Milner 2005 | | Canada | Nurses registered with the Alberta Association of registered nurses in Alberta, Canada | Subjects: **S**taff nurses, educators and managers  Characteristics:  (Educators/Staff nurses/Managers)  -Diploma (32%/70%/38%), degree (68%/30%/62%)  - Hospital (57%/67%/40%), community (18%/17%20%), LTC (5%/12%/20%), other (17%/4%/20%)  Size: N **=** 389  Response rate: 84%/90.4%/88.9% | Not reported | Mean research utilization scores  **Overall RU**  Staff: 3.63  Educator: 4.40  Managers: 3.81  **Instrumental RU**  Staff: 3.46  Educator: 4.01  Managers: 3.50  **Conceptual RU**  Staff: 3.58  Educator: 4.20  Managers: 3.77  **Symbolic RU**  Staff: 2.60  Educator: 3.50  Managers: 3.27  5-point Likert scale  1 = never  5 = very often  Refer to Estabrooks research utilization items | Moderate- Low (PRU-staff nurses)  Moderate- High  IRU (staff, managers)  CRU (staff, managers)  ORU (staff, managers)  PRU (educators, managers)  High  IRU (educators)  CRU (educators)  ORU (educators) | Strong |
| Kenny, 2005  Report 1/2 | | United States | Three hospitals in the North Atlantic Regional Medical Command | Subjects: Registered nurses  Characteristics:  Advanced practice position (10%), upper management position (6.9%); middle management (19.7%), lower management (8.6%)  -Associate degree (9.3%), diploma (4.8%), baccalaureate (56.9%), masters (26.9%), doctorate (1.4%)  -Mean yrs overall = 15.7  Size: N = 290  Response rate**:** 36.4% | Reliability:  α(subscales) = 0.75 to 0.93  Validity: Not reported | Mean research utilization    Overall: 4.52 (SD 1.85)  Direct: 4.18 (SD 1.93)  Indirect: 4.66 (SD 1.88)  Persuasive: 3.63 (SD 1.74)  7 pt frequency scale  (1) never; (5) about half the shifts; (7) nearly every shift  Refer to Estabrooks, 1997 | Moderate- Low (PRU)  Moderate-High (IRU, CRU, ORU) | Moderate - Low |
| Estabrooks, 2007  Report 2/2 | | Canada  and United States | Canada: health care settings in Alberta (mainly hospitals)  United States: three US Army hospitals in Northeast | Subjects: Nurses  Characteristics:  (US army sample/Canadian civilian sample):  -Diploma (14.2%/71.4%), baccalaureate (57.1%/25.4%), masters (27.0%/0.5%), other (1.7%/2.7%)  Size:  Canadian-N = 600  US Army-N = 290  Response rate:  Canada: 40%  United States: 34% | Reliability: Not reported  Validity: Content | Mean research utilization in the past year  **Overall**  Canada: 4.68 (SD 1.72)  United States: 4.53 (SD 1.76)  **Instrumental**  Canada: 4.47 (SD 1.92)  United States: 4.18 (SD 1.93)  Refer to Estabrooks, 1999  7-point response alternatives  1 = never  2 = on one or two shifts  3 = unlabelled  4 = unlabelled  5 = on about half of the shifts  6 = unlabelled  7 = nearly every shift  8 = do not know | Moderate-High (IRU, ORU) | Moderate - High |
| Connor, 2007 | | Canada | Nursing home facilities | Subjects: Registered nurses (n = 39), licensed practice nurses (n = 31)  Characteristics (RNs/LPNs):  **-**Diploma (79.5%/83.9%), baccalaureate (10.3%/NA for LPN), masters (2.6%/NA for LPN), Other-specialty certificate (7.7%/6.5%)  -Majority 20-30 yrs worked in nursing: (35.9%/32.3%)  -Management position (35.9%/3.2%)  Size: N = 143  Response rate: 42.9% | Reliability: Nor reported  Validity: Content by pilot study with six individuals from each of the three groups | Mean frequency of research use in the past year  **RNs:**  Direct: 4.92 (SD 1.59)  Indirect: 3.94 (SD 1.66)  Persuasive: 6.07 (SD 1.38)  Overall: (asked three times)  #1: 4.63 (SD 1.80)  #2: 5.18 (SD 1.60)  #3: 5.33 (SD 1.76)  **LPNs:**  Direct: 4.82 (SD 1.82)  Indirect: 2.80 (SD 1.42)  Persuasive: 5.27 (SD 1.62)  Overall: (asked three times)  #1: 3.64 (SD 2.02)  #2: 4.32 (SD 1.70)  #3: 3.88 (SD 1.62)  7-point response alternatives  1 = never  2 = on one or two shifts  3 = unlabelled  4 = unlabelled  5 = on about half of the shifts  6 = unlabelled  7 = nearly every shift  8 = do not know  Adapted Estabrooks 1997 | Moderate- Low (CRU)  Moderate- High (IRU, ORU)  High (PRU) | Weak |
|  | Other Single Item Measures | | | | | | | |
| Linde, 1989 | | United States | Three general surgery units | Subjects**:** staff nurses  Characteristics:  -LPN (9.7%), A.D (23.8%), Diploma (9.7%), baccalaureate (47%), masters (3.2%), Other Degree (6.5%)  -Mean age = 33 yrs  -Mean yrs in nursing **=** 7.68  Size:  Pre-185  Post- 148  Response rate:  Pre: 91:to 99%  Post: 68.5% to 85.7% | Reliability: α (current research activities, non-staff nurses, previous study) = 0.87  Validity: Pre-test with 50 nurses showed that the questions were clear | Research Activities  (3 applicable items from 6)  a) Transfer of knowledge from research into practice in the last year  none: 49.4%  once: 20.6%  two to four: 20.9%  five or more: 9.1%  b) Use of a new nursing activity based on nursing research in last year:  No activity: 41.3%  Once: 23.0%  Two to four times: 27.0%  five or moretimes: 8.7%  c) Discontinuation of a traditional activity because of the results of research  No activity: 54.8%  Once: 26.3%  Two to four times: 16.2%  Five or more times: 2.7% | Low  (item c)  Moderate- Low  (items a and b) | Moderate - Low |
| Walczak,  1994 | | United States | National Cancer  Institute-designated comprehensive cancer  centre in a mid-Atlantic metropolitan area | Subjects**:** Registered nurses (oncology)  Characteristics:  -Diploma (5%), associate degree (10%), baccalaureate (69%), masters (14%), doctorate (3%)  -Mean age = 32.5  -Years in nursing = 8.8  -Clinical nurse (37%), Senior clinical nurse (42%), Nurse manager (4%), Clinical specialist (10%), Shift coordinator (3%), Other (5%)  Size: N = 82  Response rate : 49% | Reliability: Test-retest correlation (part 3, pre-test) = 0.84  α (part 3, whole sample) = 0.88  Validity: Content (part 3)-documented by Stetler (1983, 1985), other research related literature  (American Nurses Association Commission on Nursing Research  (1981), and  the investigators' experiences | Utilizing research findings as a basis for practice  None: 10%  Low: 35%  Moderate: 29%  High: 7%  Item taken directly from Stetlers’ (1984) tool | Moderate- Low | Moderate - Low |
| Pettengill, 1994 | | United States | Members of the Midwest  Alliance in Nursing (MAIN) and Midwest Nursing Research Society | Subjects**:** Nurses- administrative, direct care, faculty, and research positions  Characteristics:  -Masters (48.5%), doctorate (23%)  Size: N = 422 (returned)  Response rate: 78% (returned) | Reliability: Not reported  Validity: Content evaluated by two nurse educators and two nurse administrators | Which of the following are you now involved in related to using nursing research findings in your practice?  Apply non-nursing research  Service Group (n = 222) = 62%  Implement by self  Service Group = 38% | Moderate- Low  (use of nursing research)  Moderate- High  (use of non-nursing research) | Weak |
| Veeramah, 1995 | | England | Specific clinical areas in the south-east of England | Subjects**:** Nurses  Characteristics:  Not reported  Size: N = 118  Response rate: 78% | Reliability: Not reported  Validity: Pilot tested with  Research Interest  Group Nurses –  Don’t report number  or whether they evaluated the content | Nursing research findings used in one’s area to improve patient/client care  Great extent: 15.3%  Some extent: 55.1%  Little extent: 22.9%  Not at all: 3.4% | Moderate- High | Moderate - Low |
| Youngstrom 1996 | | United States | National sample of hospitals accredited by the Joint Commission on Accreditation of Healthcare Organizations (JCAHO) | Subjects**:** Nursing staff development educators  Characteristics:  -Associate degree (5.1%), diploma (5.3%)  LPN (0.2%), baccalaureate (35.2%)  masters (50.3%), doctorate (2.7%)  **-**Mean age = 43.7yrs  -Mean yrs in nursing = 20.7  Size: N = 531  Response rate: 41% | Reliability: α (stages of adoption decision sub-scale) = 0.92.  Test retest r = 0.87  Validity: Instrument based on current literature. Pilot tested to ask respondents about understandability.  Construct-Factor analysis | Integrate research results into educational activities:  Unaware (of the need to integrate research results): 8.3%  *Those aware were then asked to choose one of the following options*  Persuaded (believe it is important to integrate research results): 22.8%  Decided (decided to integrate research results): 5.3%  Adopted (Do integrate research results): 23.7%  Confirmed (intend to continue to integrate research results): 39.9% | Moderate-High  (adopted 23.7% + confirmed 39.9% = 63.3% using) | Weak |
| Wright, 1996 | | Australia | Hospitals and community mental health centers | Subjects: Registered nurses-general and psychiatric  Characteristics:  -Baccalaureate (60.5%)  -Mean age = 36yrs  Size: N = 410  Response rate: 82% | Reliability: Not reported Validity: Content by consultation with three clinical nurse consultants | Findings (from research) applied to patient care: 60% | Moderate- High | Weak |
| Logsdon, 1998 | | United States | Kentucky | Subjects: Nurses registered with the Kentucky Board of Nursing  Characteristics:  -Associate degree (54.6%)  Size: N = 196  Response rate: 20% | Reliability: Not reportedValidity: The instrument was based on the literature and the investigators experience with RU in the clinical setting. | Utilize research findings to change their practice at least once a year = 43% | Moderate- Low | Moderate - Low |
| Davies, 1999 | | United Kingdom | 11 health authorities | Subjects**:** Practice nurses  Characteristics:  -Age between 30 and 54 (86%)  -F grade (31.7%) or G grade (58.7%)  Size: N = 1187  Response rate: 60.4% | Reliability: Not reported  Validity: Literature reviewed to identify interventions with a sound research base | 23 Interventions to prevent CVD and stroke:  Number of interventions used by individual nurses: Range = 48% to 96% | Moderate- Low/ Moderate- High/ High (depending of the specific practice) | Weak |
| Tsai, 2000 | | Republic of China | Large medical center | Subjects: Registered Nurses and Nurse Managers  Characteristics:  **-**Graduate degree (4%), baccalaureate (30%), diploma (61%), vocational qualification (5%)  -5 to 10 yrs in nursing (23%), >20yrs (20%)  Size: N = 382 (registered nurses [n = 271) and managers (n = 111)]  Response rate: Registered Nurses-68%; Nurse Managers: 63% | Reliability: Not reported  Validity: Content by expert panel and pilot test | Research use in past three years: 50%  Refer to Funk 1991 and Pettengill 1994 | Moderate- High | Moderate - High |
| Tsai 2003 | | Taiwan | One medical center | Subjects: Nurses  Characteristics (experimental andcontrol): (NS)  **-**University education (62.9%)  -Mean age = 33.9 yrs  **-**Mean yrs in nursing = 10  Size: N = 89  Control group n = 42  Experimental group n = 47  Response rate: 84.8%  (15.2% drop outs) | Reliability: Not reported  Validity: Content of tool  checked and confirmed  by five clinical nurses | Research findings used for practice over the past three years  (NS)  Experimental group:  Pretest: 46.8%  Second measure (after the course): 42.6%  Third measure (6 months after course): 51.1%  Control group:  Pretest: 42.9%  Second measure: 40.5%  Third measure: 57.1%  Adopted from Funk 1991 and Pettengill 1994 | Moderate- High | Moderate - Low |
| Niederhauser 2005 | | United States | Various clinical areas | Subjects: Paediatric nurse practitioners with NAPNAP membership  Characteristics:  - PhD (2%), masters (92%), PNP certification (6%), baccalaureate (0.3%)  - Mean age = 36.2yrs  Size: N = 396 (usable surveys)  Response rate: 69% (N = 431 total surveys) | Reliability: Not reported  Validity: Not reported | Applied research findings in practice: 79.9% | High | Moderate - Low |

^1^Extent Calculations

- Past, Present, Future Use (Bostrom 1993; Rizzuto 1994; Butler 1995; Brown 1997). 0 to 100% range. Extent calculated by dividing score range into 4 equal quartiles as follows: low (0% to 24.99%), moderate-low (25.00% to 49.99%), moderate-high (50.00% to 74.99%), high (75.00% to 100%)
- Parahoo measure (Parahoo 1998; Parahoo 199a; Parahoo 1999b; Parahoo 2000). 5 point scale (never, seldom, sometimes, frequently, all the time) assigned scale points of 1 to 5. Percentage reporting each category was given in the article, we obtained a n value for each category by multiplying by total N. Mean score on 1 to 5 scale then obtained by summing (n for each scale category X associated scale point) and taking the average. Extent calculated by dividing 1 to 5 subscale score range into 4 equal quartiles as follows: low (1.00 to 1.99), moderate-low (2.00 to 2.99), moderate-high (3.00 to 3.99), high (4.00 to 5.00)
- Parahoo measure (Parahoo 2001, Valizadeh 2003; Veeramah 2004). 3 point scale (descriptors vary, see table) assigned scale points of 1 to 3. Percentage reporting each category was given in the article; we obtained a n value for each category by multiplying by total N. Mean score on 1 to 3 scale then obtained by summing (n for each scale category X associated scale point) and taking the average. Extent calculated by dividing 1 to 3 subscale score range into 4 equal quartiles as follows: low (1.00 to 1.49), moderate-low (1.50 to 1.99), moderate-high (2.00 to 2.49), high (2.50 to 3.00)
- Estabrooks’ Kinds of research use. 5 articles (Estabrooks 1999; Profetto-McGrath 2003; Kenny 2005; Estabrooks 2007, Connor 2007) used a 1 to 7 scale. Extent calculated by dividing score range into 4 equal quartiles as follows: low (1 to 2.49), moderate-low (2.50 to 3.99), moderate-high (4.00 to 5.49), high (5.50 to 7.00). 1 article (Milner 2005) used a 1 to 5 scale. Extent calculated by dividing 1 to 5 subscale score range into 4 equal quartiles as follows: low (1.00 to 1.99), moderate-low (2.00 to 2.99), moderate-high (3.00 to 3.99), high (4.00 to 5.00)
- Other single items (Linde 1989; Walczak 1994; Veeramah 1995). 1 to 4 scale range. Extent calculated by dividing mean scale score range into 4 equal quartiles as follows: low (1.00 to 1.74), moderate-low (1.75 to 2.49), moderate-high (2.50 to 3.24), high (3.25 to 4.00)
- Other single items (Pettengill 1994; Youngstrom 1996; Wright 1996; Logsdon 1998; Davies 1999; Tsai 2000; Tsai 2003; Niederhauser 2005). 0 to 100% range. Extent calculated by dividing score range into 4 equal quartiles as follows: low (0% to 24.99%), moderate-low (25.00% to 49.99%), moderate-high (50.00% to 74.99%), high (75.00% to 100%)
