# Supplementary material for: To what extent do nurses use research in clinical practice? A systematic review
Source: Implement Sci. 2011 Mar 17;6:21. doi: 10.1186/1748-5908-6-21 (PMC3068972; doi:10.1186/1748-5908-6-21)
Supplement: Additional file 5 — Excluded Articles based on Full-Text (n = 78). Citation and reason for exclusion for 78 articles retrieved but not included in the review. [file 1748-5908-6-21-S5.DOC]

**Additional File 5. Excluded Articles based on Full-Text (n = 78)**

| **Citation** | **Exclusion Reason** |
| --- | --- |
| Abouzelof, R. H. (1999). Diffusion of innovations: Describing the perceptions of the stages in the innovation-decision process for handwashing and alcohol hand rubs. (M.S.N., University of Utah College of Nursing). | Article purpose is to record handwashing diffusion, not to measure research use |
| Adamsen, L., Larsen, K., Bjerregaard, L., and Madsen, J. K. (2003). Danish research-active clinical nurses overcome barriers in research utilization. Scandinavian Journal of Caring Sciences, 17(1), 57-65. | Determinants of research use and use of research |
| Anderson, M., Cosby, J., Swan, B., Moore, H., and Broekhoven, M. (1999). The use of research in local health service agencies. Social Science and Medicine, 49(8), 1007-1019. | About concept analysis |
| Baessler, C. A., Blumberg, M., Cunningham, J. S., Curran, J. A., Fennessey, A. G., Jacobs, J. M., et al. (1994). Medical-surgical nurses' utilization of research methods and products. Medsurg nursing: Official journal of the Academy of Medical-Surgical Nurses, 3(2), 113-117, 120. | Sources of knowledge |
| Bookbinder, M. I. (1992). Nurse linkage agents' efforts to facilitate the use of a research-based innovation. (PH.D., New York University). | Purpose is to measure use of specific practice, not to measure research use |
| Bostrom, A., Wallin, W., Nordstrom, G. (2006). Research use in the care of older people: A survey among healthcare staff. International Journal of Older People Nursing, 1(3), 131-140. | Did not measure research use in nurses separately from other care providers |
| Burkoski, V. (2002). Infant sleep position: Nurses' awareness and practice of the Canadian joint statement recommendation. (M.Sc., University of Windsor) | Measuring guideline use |
| Camiah, S. (1997). Utilization of nursing research in practice and application of strategies to raise research awareness amongst nurse practitioners: A model for success. Journal of Advanced Nursing, 26(6), 1193-1202. | Qualitative |
| Camiletti, Y. A., and Huffman, M. C. (1998). Research utilization: Evaluation of initiatives in a public health nursing division. Canadian Journal of Nursing Administration, 11(2), 59-77. | Valuing research |
| Capra. (1992). RNs utilization of research findings. The American Journal of Advanced Nursing, 10(1), 21-25. | Adherence to specific practice (fasting) |
| Clifford, C., and Murray, S. (2001). Pre- and post-test evaluation of a project to facilitate research development in practice in a hospital setting. Journal of Advanced Nursing, 36(5), 685-695. | Measures factors related to attitudes |
| Crane, J. (1989). Factors associated with the use of research-based knowledge in nursing (Dissertation). | Measures use of knowledge packages, partly research based. Also participation in research activities, items not described |
| Crawford, V. L., McPeake, B., and Stout, R. W. (1995). Diagnostic regimes for urinary tract infection--are research results applied to practice? Ulster Medical Journal, 64(2), 131-136. | Nurses not discussed specifically |
| Davies, B. L. (1999). Evaluation of two strategies for the transfer of research results about labour support and electronic fetal monitoring into practice. (Ph.D., University of Toronto, Canada). | Is about a dissemination strategy |
| Dennis, A. R., Leeson-Payne, C. G., Langham, B. T., and Aitkenhead, A. R. (1995). Local anaesthesia for cannulation. Has practice changed? see comment. Anaesthesia, 50(5), 400-402. | Not nurses |
| Doerflinger, D. M. (2004). The relationship between acute care nurse administrators' knowledge and attitudes and restraint reduction. (Ph.D., George Mason University). | Adherence to specific practice (restraint use) |
| Dufault MA, Bielecki C, Collins E, Willey C. (1995). Changing nurses' pain assessment practice: a collaborative research utilization approach. Journal of Advanced Nursing. 21(4):634-45. | Research use not measured |
| Eller, L. S., Kleber, E., and Wang, S. L. (2003). Research knowledge, attitudes and practices of health professionals. Nursing Outlook, 51(4), 165-170. | Research use not measured; measured knowledge, attitudes and practice |
| Estabrooks, C. A. (1999). Modeling the individual determinants of research utilization. Western Journal of Nursing Research, 21(6), 758-772. | About determinants of research use |
| Estabrooks, C. A., Chong, H., Brigidear, K., and Profetto-McGrath, J. (2005). Profiling Canadian nurses' preferred knowledge sources for clinical practice. Canadian Journal of Nursing Research, 37(2), 118-140. | About sources of knowledge |
| Ferlie, E., Fitzgerald, L., and Wood, M. (2000). Getting evidence into clinical practice: An organizational behaviour perspective. Journal of Health Services and Research Policy, 5(2), 96-102. | The only portion specific to nurses is not research based |
| Forbes, S. A., Bott, M. J., and Taunton, R. L. (1997). Control over nursing practice: A construct coming of age. Journal of Nursing Measurement, 5(2), 179-190. | Only psychometrics |
| Frantz, R. A., Gardner, S., Harvey, P., and Specht, J. (1992). Adoption of research-based practice for treatment of pressure ulcers in long-term care. Decubitus, 5(1), 44-5, 48-50, 52. | Nursing practice not discussed specifically |
| Freeman, C.K. (1993). Breastfeeding care in Ohio hospitals: A gap between research and practice. JOGNN Clinical Studies, 22(5), 447-452. | Wrong unit of analysis (hospital level) |
| Goode, C. J., Lovett, M. K., Hayes, J. E., and Butcher, L. A. (1987). Use of research based knowledge in clinical practice. Journal of Nursing Administration, 17(12), 11-18. | Research use not measured |
| Grap, M. J., Pettrey, L., and Thornby, D. (1997). Hemodynamic monitoring: a comparison of research and practice. American Journal of Critical Care, 6(6), 452-456. | Compliance with a single, specific practice |
| Hammond, A., and Klompenhouwer, P. (2005). Getting evidence into practice: Implementing a behavioural joint protection education programme for people with rheumatoid arthritis. British Journal of Occupational Therapy, 68(1), 25-33. | Purpose is to examine use of a specific research-based practice |
| Happell, B., and Martin, T. (2004). Exploring the impact of the implementation of a nursing clinical development unit program: What outcomes are evident? International Journal of Mental Health Nursing, 13(3), 177-184. | Research use not measured |
| Harris, M. (1992). The impact of research findings on current practice in relieving postpartum perineal pain in a large district general hospital. Midwifery, 8(3), 125-131. | Doctors and midwives analyzed together |
| Hart, G. M. (1988). Change theory and the impact of an educational program on the case-finding activities of registered nurses in the early identification of alcohol-related problems. (Rutgers the State University of New Jersey-New Brunswick) | Is about the impact of an educational intervention |
| Helberg, D., Mertens, E., Halfens, R. J., and Dassen, T. (2006). Treatment of pressure ulcers: Results of a study comparing evidence and practice. Ostomy Wound Management, 52(8), 60-72. | Nurses research use not specified |
| Hoffart, N., and Cobb, A. K. (2002). Assessing clinical pathways use in a community hospital: It depends on what "use" means. The Joint Commission Journal on Quality Improvement, 28(4), 167-179. | Clinical pathway use |
| Hong, S. W., and Ching, T. Y. 1990. The employment of ward opinion leaders for continuing education in the hospital. Medical Teacher*,* 12(2), 209. | Measures guideline adherence |
| Howell, S. L., Foster, R. L., Hester, N. O., Vojir, C. P., and Miller, K. L. (1996). Evaluating a pediatric pain management research utilization program. Canadian Journal of Nursing Research, 28(2), 37-57. | Specific practice (pain management); program evaluation |
| Johnson, F. E., and Maikler, V. E. (2001). Nurses' adoption of the AWHONN/NANN neonatal skin care project. Newborn and Infant Nursing Reviews, 1(1), 59-67. | Measures use of a protocol which is not necessarily research based |
| Kelly, J. A., Somlai, A. M., DiFranceisco, W. J., Otto-Salaj, L. L., McAuliffe, T. L., Hackl, K. L., et al. (2000). Bridging the gap between the science and service of HIV prevention: Transferring effective research-based HIV prevention interventions to community AIDS service providers. American Journal of Public Health, 90(7), 1082-1088. | Research use not measured; purpose is to measure model adoption |
| Kenrick, M., and Luker, K. A. (1996). An exploration of the influence of managerial factors on research utilization in district nursing practice. Journal of Advanced Nursing, 23(4), 697-704. | Factors related to research use |
| Ketefian, S. (1975) Application of selected nursing research findings into nursing practice: A pilot study. Nursing Research, 24(2) 89-92. | Compliance with a single, specific practice |
| Kirchhoff, K. T. (1982). A diffusion survey of coronary precautions. Nursing Research, 31(4), 196-201. | Compliance with a single, specific practice |
| Kothari, A., Birch, S., and Charles, C. (2005). "Interaction" and research utilisation in health policies and programs: Does it work? Health Policy, 71(1), 117-125. | Research use not measured |
| Krueger, J. C. (1982). Using research in practice: A survey of research utilization in community health nursing. Western Journal of Nursing Research, 4(2), 244-248. | Managers not staff nurses |
| Lacey, E. A. (1996). Facilitating research-based practice by educational intervention. Nurse Education Today, 16(4), 296-301. | Research use not measured. Instrument is used to evaluate the extent to which an educational intervention can influence practice |
| Lia-Hoagberg, B., Schaffer, M., and Strohschein, S. (1999). Public health nursing practice guidelines: An evaluation of dissemination and use. Public Health Nursing, 16(6), 397-404. | Measures guideline use |
| LoftusHills, A., and Duff, L. (1997). Implementation of nutrition standards for older adults. Nursing Standard, 11(44), 33-37. | Measures guideline adherence |
| McCleary, L., and Brown, G. T. (2002). Research utilization among pediatric health professionals. Nursing and Health Sciences, 4(4), 163-171. | Nurses and allied health professionals analyzed and reported upon together |
| McCleary, L., and Brown, G. T. (2003). Barriers to paediatric nurses' research utilization. Journal of Advanced Nursing, 42(4), 364-372. | Discussion of barriers but does not report on extent of research use |
| McCleary, L., Ellis, J. A., and Rowley, B. (2004). Evaluation of the pain resource nurse role: a resource for improving pediatric pain management. Pain Management Nursing, 5(1), 29-36. | About how a program influenced a specialists' role |
| Miller, L. M., and Nugent, K. P. (2003). Surgical integrated care pathway development: Compliance and staff satisfaction. Journal of Integrated Care Pathways, 7(1), 36-46. | Article purpose is not to measure research use |
| Molassiotis, A. (1997). Nursing research within bone marrow transplantation in Europe: An evaluation. European Journal of Cancer Care, 6(4), 257-261. | Qualitative |
| Montgomery, L. A., Hanrahan, K., Kottman, K., Otto, A., Barrett, T., and Hermiston, B. (1999). Guideline for I.V. infiltrations in pediatric patients. Pediatric Nursing, 25(2), 167-169. | Measures guideline compliance |
| Murtaugh, C. M., Pezzin, L. E., McDonald, M. V., Feldman, P. H., and Peng, T. R. (2005). Just-in-time evidence-based e-mail "reminders" in home health care: Impact on nurse practices. Health Services Research, 40(3), 849-864. | About cost |
| Nelson, D. (1995). Research into research practice. Accident and Emergency Nursing, 3(4), 184-189. | Research use not measured; rather, is about the action plan and implementing the action plan activity |
| Olade, R. A. (2004). Evidence-based practice and research utilization activities among rural nurses. Journal of Nursing Scholarship, 36(3), 220-225. | Qualitative |
| Pepler, C. J., Edgar, L., Frisch, S., Rennick, J., Swidzinski, M., White, C., et al. (2005). Unit culture and research-based nursing practice in acute care. Canadian Journal of Nursing Research, 37(3), 66-85. | Qualitative |
| Pain, K., Hagler, P., and Warren, S. (1996). Development of an instrument to evaluate the research orientation of clinical professionals. Canadian Journal of Rehabilitation, 9(2), 93-100. | RNs were not analyzed separately |
| Panagiotopoulou, K., and Kerr, S. M. (2002). Pressure area care: An exploration of Greek nurses' knowledge and practice. Journal of Advanced Nursing, 40(3), 285-296. | Article purpose is not to measure research use |
| Parkin, C., and Bullock, I. (2005). Evidence-based health care: Development and audit of a clinical standard for research and its impact on an NHS trust. Journal of Clinical Nursing, 14(4), 418-425. | Lack of clarity and detail in reporting |
| Pennington, L. (2001). Attitudes to and use of research in speech and language therapy. British Journal of Therapy and Rehabilitation, 8(10), 375-6, 378-9. | Not nurses |
| Rangeley, H., and Arthurs, J. (2004). The long-term effects of undertaking a research course on clinical practice. Nurse Education in Practice, 4(1), 12-19. | Report why they use research, not how much or how often. |
| Reynolds, M. I. A. (1981). An investigation of organizational factors affecting research utilization in nursing organizations (Dissertation) | Research use of nurses specifically not measured |
| Riegel, B., Thomason, T., Carlson, B., and Gocka, I. (1996). Are nurses still practicing coronary precautions? A national survey of nursing care of acute myocardial infarction patients. American Journal of Critical Care, 5(2), 91-98. | Compliance with a single, specific practice |
| Ring, N., Malcolm, C., Coull, A., Murphy-Black, T., and Watterson, A. (2005). Nursing best practice statements: an exploration of their implementation in clinical practice. Journal of Clinical Nursing, 14(9), 1048-1058. | Qualitative; Measures guideline (BPS) use |
| Ring, N., Coull, A., Howie, C., Murphy-Black, T., and Watterson, A. (2006). Analysis of the impact of a national initiative to promote evidence-based nursing practice. International Journal of Nursing Practice. 12(4), 232-240. | Measures guideline (BPS) use |
| Scott, S. D. et. al. (2008). A context of uncertainty: How context shapes nurses' research utilization behaviors. Qualitative Health Research, 18(3), 347-357. | Research use not measured |
| Specht, J. P., Bergquist, S., and Frantz, R. A. (1995). Adoption of a research-based practice for treatment of pressure ulcers. Nursing Clinics of North America, 30(3), 553. | Research use not measured |
| Sproat, L. J., and Inglis, T. J. (1994). A multicentre survey of hand hygiene practice in intensive care units. Journal of Hospital Infection, 26(2), 137-148. | Compliance with a single, specific practice |
| Stetler, C. B., and DiMaggio, G. (1991). Research utilization among clinical nurse specialists. *Clinical Nurse Specialist,* 5(3), 151-155. | Not able to determine extent of research use |
| Suter, E., Vanderheyden, L. C., Trojan, L. S., Verhoef, M. J., and Armitage, G. D. (2007). How important is research-based practice to chiropractors and massage therapists? Journal of Manipulative and Physiological Therapeutics, 30(2), 109-115. | Not nurses |
| Valente, S. M. (2005). Evaluation of innovative research-based fact sheets. Journal for Nurses in Staff Development, 21(4), 171-176. | Study purpose is to measure use of fact sheets, not to measure level of research use |
| Van Mullem, C., Burke, L. J., Dohmeyer, K., Farrell, M., Harvey, S., John, L., et al. (1999). Strategic planning for research use in nursing practice. Journal of Nursing Administration, 29(12), 38-45. | Measures willingness but not actual research use |
| Wallin, L., Estabrooks, C. A., Midodzi, W. K., and Cummings, G. G. (2006). Development and validation of a derived measure of research utilization by nurses. Nursing research, 55(3), 149-160. | No measure research use. Research use variable is derived based on predictors |
| White, C. L. (1999). Changing pain management practice and impacting on patient outcomes. Clinical Nurse Specialist, 13(4), 166-172. | Research use not measured |
| Williams, E., and Buckles, A. (1988). A lack of motivation. Nursing times. 84(22), 60 -64. | Research use not measured |
| Williams, E. E. (1987) Psychological contributions to the control of hospital acquired infections. (Dissertation.) | Research use not measured |
| Winter, J. C. (1990). Relationship between sources of knowledge and use of research findings. Journal of Continuing Education in Nursing, 21(3), 138-140. | Compliance with a single, specific practice |
| Won, S., Chou, H., Hsieh, W., Chen, C., Huang, S., Tsou, K., et al. (2004). Handwashing program for the prevention of nosocomial infections in a neonatal intensive care unit. Infection Control and Hospital Epidemiology, 25(9), 742-746. | Research use not measured |
| Wygnanski-Jaffe, T. (2005). The effect on pediatric ophthalmologists of the randomized trial of patching regimens for treatment of moderate amblyopia. Journal of AAPOS: American Association for Pediatric Ophthalmology and Strabismus, 9(3), 208-211. | Not nurses |
