# Supplementary material for: To what extent do nurses use research in clinical practice? A systematic review
Source: Implement Sci. 2011 Mar 17;6:21. doi: 10.1186/1748-5908-6-21 (PMC3068972; doi:10.1186/1748-5908-6-21)
Supplement: Additional file 6 — Quality Assessment. A summary of quality assessment of the 55 articles included in the review. [file 1748-5908-6-21-S6.DOC]

**Additional file 6. Quality Assessment**

Quality Assessment for the Included Cross Sectional Articles (n = 51)

| **First Author, year** | **Sample** | | | | | | | **Measurement** | | | **Statistical Analysis** | | | | **Total Points^1^** | **Score** | **Quality** |
| --- | --- | --- | --- | --- | --- | --- | --- | --- | --- | --- | --- | --- | --- | --- | --- | --- | --- |
|  | Probabilistic sample used | Representative | Sample size appropriate for power | Sample drawn >1 site | Matching design | Statistically adjusted | Response rate >50% | DV measurement | DV reliability | DV validity | Appropriate tests used | p values reported | CI values reported | Missing data managed appropriately |  |  |  |
| Pettengill, 1994 | 0 | 1 | 1 | 1 | 0 | 0 | 1 | 0 | 0 | 1 | 0 | 0 | 0 | 0 | 5/16 | 0.31 | Weak |
| Connor, 2006 | 0 | 1 | 0 | 1 | 0 | 0 | 0 | 0 | 0 | 1 | 1 | 1 | 0 | 1 | 6/16 | 0.38 | Weak |
| Butler, 1995 | 0 | 1 | 1 | 0 | 0 | 0 | 1 | 0 | 1 | 0 | 1 | 1 | 0 | 1 | 7/16 | 0.44 | Weak |
| Wright, 1996 | 0 | 1 | 1 | 1 | 0 | 0 | 1 | 0 | 0 | 1 | 1 | 1 | 0 | 0 | 7/16 | 0.44 | Weak |
| Hansen, 1999 | 0 | 1 | 0 | 0 | 0 | 0 | 1 | 0 | 1 | 1 | 1 | 1 | 0 | 1 | 7/16 | 0.44 | Weak |
| McCloskey, 2005 | 0 | 0 | 1 | 1 | N/A | N/A | 0 | 0 | 1 | 1 | 1 | 1 | 0 | 0 | 6/13 | 0.46 | Weak |
| Parahoo, 1999 | 0 | 1 | 1 | 1 | N/A | N/A | 1 | 0 | 0 | 1 | 0 | N/A | N/A | 0 | 5/11 | 0.45 | Weak |
| Rizzuto, 1994 | 0 | 0 | 1 | 1 | N/A | N/A | 0 | 0 | 1 | 1 | 1 | N/A | N/A | 0 | 5/11 | 0.45 | Weak |
| Champion, 1989 | 0 | 1 | 1 | 0 | N/A | N/A | 0 | 0 | 1 | 1 | 1 | 1 | 0 | 0 | 6/13 | 0.46 | Weak |
| Lacey, 1994 | 1 | 1 | 0 | 1 | N/A | N/A | 0 | 0 | 0 | 1 | 1 | 1 | 0 | 0 | 6/13 | 0.46 | Weak |
| Nash, 2005 | 1 | 0 | 1 | 1 | N/A | N/A | 0 | 0 | 1 | 1 | 1 | 0 | 0 | 0 | 6/13 | 0.46 | Weak |
| McCleary, 2003 | 0 | 1 | 1 | 0 | N/A | N/A | 0 | 0 | 1 | 1 | 1 | 1 | 0 | N/A | 6/12 | 0.50 | Weak |
| Youngstrom, 1996 | 1 | 1 | 1 | 1 | 0 | 0 | 0 | 0 | 1 | 1 | 1 | 1 | 0 | 0 | 8/16 | 0.50 | Weak |
| Davies, 1999 | 0 | 1 | 1 | 1 | N/A | N/A | 1 | 0 | 1 | 1 | 0 | 1 | N/A | 0 | 6/12 | 0.50 | Weak |
| Stiefel, 1996 | 1 | 1 | 1 | 1 | 0 | 0 | 0 | 0 | 1 | 1 | 1 | 1 | 0 | 0 | 8/16 | 0.50 | Weak |
| Bostrom, 1993 | 0 | 1 | 1 | 1 | N/A | N/A | 0 | 0 | 1 | 1 | 1 | 0 | N/A | 0 | 6/12 | 0.50 | Weak |
| Brown, 1997 | 0 | 1 | 1 | 1 | N/A | N/A | 1 | 0 | 0 | 1 | 1 | 1 | 0 | 0 | 7/13 | 0.54 | Moderate-Low |
| McCleary, 2002 | 0 | 1 | 1 | 0 | N/A | N/A | 0 | 0 | 1 | 1 | 1 | 1 | 0 | 1 | 7/13 | 0.54 | Moderate-Low |
| Parahoo, 1999 | 0 | 1 | 1 | 1 | N/A | N/A | 1 | 0 | 0 | 1 | 1 | 1 | 0 | 0 | 7/13 | 0.54 | Moderate-Low |
| Parahoo, 2001 | 0 | 1 | 1 | 1 | N/A | N/A | 1 | 0 | 0 | 1 | 1 | 1 | 0 | 0 | 7/13 | 0.54 | Moderate-Low |
| Rutledge, 1996 | 0 | 1 | 1 | 1 | N/A | N/A | 0 | 0 | 1 | 1 | 1 | 1 | 0 | 0 | 7/13 | 0.54 | Moderate-Low |
| Wallin, 2003 | 0 | 2 | 0 | 0 | N/A | N/A | 1 | 0 | 1 | 1 | 1 | 1 | 0 | 0 | 7/13 | 0.54 | Moderate-Low |
| Profetto-McGrath, 2003 | 0 | 1 | 1 | 1 | N/A | N/A | 0 | 0 | 1 | 1 | 1 | 1 | 0 | 0 | 7/13 | 0.54 | Moderate-Low |
| Parahoo, 1998 | 0 | 1 | 1 | 1 | N/A | N/A | 1 | 0 | 0 | 1 | 1 | N/A | N/A | 0 | 6/11 | 0.55 | Moderate-Low |
| Parahoo, 2000 | 0 | 1 | 1 | 1 | N/A | N/A | 1 | 0 | 0 | 1 | 1 | N/A | N/A | 0 | 6/11 | 0.55 | Moderate-Low |
| Veeramah, 1995 | 0 | 1 | 1 | 1 | N/A | N/A | 1 | 0 | 0 | 1 | 1 | N/A | N/A | 0 | 6/11 | 0.55 | Moderate-Low |
| Walczak, 1994 | 0 | 1 | 1 | 0 | N/A | N/A | 0 | 0 | 1 | 1 | 1 | N/A | N/A | 1 | 6/11 | 0.55 | Moderate-Low |
| Humphris, 1999 | 1 | 2 | 1 | 1 | 0 | 0 | 1 | 0 | 0 | 1 | 1 | 1 | 0 | 0 | 9/16 | 0.57 | Moderate-Low |
| Logsdon, 1998 | 1 | 1 | 1 | 1 | N/A | N/A | 0 | 0 | 0 | 1 | 1 | 1 | N/A | 0 | 7/12 | 0.58 | Moderate-Low |
| Veeramah, 2004 | 0 | 1 | 1 | 1 | N/A | N/A | 1 | 0 | 0 | 1 | 1 | 1 | N/A | 0 | 7/12 | 0.58 | Moderate-Low |
| Carlson, 2006 | 0 | 1 | 1 | 1 | N/A | N/A | 0 | 0 | 1 | 1 | 1 | 1 | 0 | 1 | 8/13 | 0.61 | Moderate-Low |
| Hatcher, 1997 | 1 | 2 | 1 | 0 | N/A | N/A | 0 | 0 | 1 | 1 | 1 | 1 | 0 | 0 | 8/13 | 0.62 | Moderate-Low |
| Prin, 1997 | 0 | 1 | 1 | 0 | N/A | N/A | 0 | 0 | 1 | 1 | 1 | 1 | 0 | 1 | 8/13 | 0.62 | Moderate-Low |
| Kenny, 2005 | 0 | 1 | 1 | 1 | N/A | N/A | 0 | 0 | 1 | 1 | 1 | 1 | 0 | 1 | 8/13 | 0.62 | Moderate-Low |
| Varcoe, 1995 | 1 | 1 | 1 | 1 | N/A | N/A | 0 | 0 | 1 | 1 | 1 | 1 | 0 | 0 | 8/13 | 0.62 | Moderate-Low |
| Niederhauser, 2005 | 1 | 2 | 1 | 1 | N/A | N/A | 1 | 0 | 0 | 0 | 1 | 1 | 0 | 0 | 8/13 | 0.62 | Moderate-Low |
| Berggren, 1996 | 0 | 1 | 1 | 1 | N/A | N/A | 1 | 0 | 1 | 1 | 1 | N/A | N/A | 0 | 7/11 | 0.64 | Moderate-Low |
| Michel, 1995 | 1 | 1 | 1 | 1 | N/A | N/A | 1 | 0 | 1 | 1 | 1 | 1 | 0 | 0 | 9/13 | 0.69 | Moderate-High |
| Thompson, 1997 | 1 | 1 | 1 | 1 | N/A | N/A | 0 | 0 | 1 | 1 | 1 | 1 | 0 | 1 | 9/13 | 0.69 | Moderate-High |
| Estabrooks, 1999 | 1 | 2 | 1 | 1 | N/A | N/A | 0 | 0 | 0 | 1 | 1 | 1 | 0 | 1 | 9/13 | 0.69 | Moderate-High |
| Estabrooks, 2007 | 1 | 2 | 1 | 1 | 0 | 1 | 0 | 0 | 1 | 1 | 1 | 1 | 1 | 1 | 12/16 | 0.75 | High  Moderate |
| Tsai, 2000 | 1 | 2 | 1 | 0 | N/A | N/A | 1 | 0 | 1 | 1 | 1 | 1 | N/A | 0 | 9/12 | 0.75 | Moderate-High |
| Barta, 1995 | 0 | 1 | 1 | 1 | N/A | N/A | 1 | 0 | 1 | 1 | 1 | 1 | N/A | 1 | 9/12 | 0.75 | Moderate-High |
| Brett, 1987 | 1 | 2 | 1 | 1 | N/A | N/A | 1 | 0 | 1 | 1 | 1 | 1 | 0 | 0 | 10/13 | 0.77 | Moderate-High |
| Brett, 1989 | 1 | 2 | 1 | 1 | N/A | N/A | 1 | 0 | 1 | 1 | 1 | 1 | 0 | 0 | 10/13 | 0.77 | Moderate-High |
| Coyle, 1990 | 1 | 2 | 1 | 1 | N/A | N/A | 1 | 0 | 1 | 1 | 1 | 1 | 0 | 0 | 10/13 | 0.77 | Moderate-High |
| Squires, 2007 | 0 | 1 | 1 | 1 | N/A | N/A | 1 | 0 | 1 | 1 | 1 | 1 | 1 | 1 | 10/13 | 0.77 | Moderate-High |
| Rodgers, 2000 | 1 | 2 | 1 | 1 | N/A | N/A | 1 | 0 | 1 | 1 | 1 | 1 | 0 | 1 | 11/13 | 0.85 | Moderate-High |
| Valizadeh, 2003 | 1 | 2 | 1 | 1 | N/A | N/A | 1 | 0 | 1 | 1 | 1 | N/A | N/A | 0 | 9/11 | 0.82 | Strong |
| Milner, 2005 | 1 | 2 | 1 | 1 | 2 | N/A | 1 | 0 | 1 | 1 | 1 | 1 | 0 | 1 | 13/15 | 0.87 | Strong |
| Rodgers, 2000 | 1 | 2 | 1 | 1 | N/A | N/A | 1 | 0 | 1 | 1 | 1 | N/A | N/A | 1 | 10/11 | 0.91 | Strong |

^1^Total Points = 15 total points possible; DV = Dependent Variable; CI = Confidence Interval

Weak (≤0.50), Moderate-Low (0.51 to 0.65), Moderate-High (0.66 to 0.79), or Strong (≥0.80)

Quality Assessment for the Included Quasi-Experimental Articles (n = 4)

| **First Author, Year** | **Selection Bias** | **Allocation Bias** | **Confounders** | **Blinding** | **Data Collection Methods** | **Withdrawals and Drop-Outs** | **Total Points^1^** | **Score** | **Quality** |
| --- | --- | --- | --- | --- | --- | --- | --- | --- | --- |
| Pelz, 1981 | Weak | Moderate | Weak | Not applicable | Weak | Weak | 6/5 | 1.2 | Weak |
| Linde, 1989 | Moderate | Moderate | Weak | Not applicable | Moderate | Weak | 8/5 | 1.6 | Moderate-Low |
| Tsai, 2003 | Weak | Moderate | Weak | Not applicable | Moderate | Strong | 9/5 | 1.8 | Moderate-Low |
| Tranmer, 2002 | Moderate | Moderate | Strong | Not applicable | Strong | Weak | 11/5 | 2.2 | Moderate-High |

Weak (1 to 1.5), Moderate-Low (1.6 to 2.0), Moderate-High (2.1 to 2.5), or Strong (>2.5)
